# Effects of Kisspeptin on Sexual Brain Processing and Penile Tumescence in Men With Hypoactive Sexual Desire Disorder: A Randomized Clinical Trial

**DOI:** 10.1001/jamanetworkopen.2022.54313

**Published:** 2023-02-03

**Authors:** Edouard G. Mills, Natalie Ertl, Matthew B. Wall, Layla Thurston, Lisa Yang, Sofiya Suladze, Tia Hunjan, Maria Phylactou, Bijal Patel, Beatrice Muzi, Dena Ettehad, Paul A. Bassett, Jonathan Howard, Eugenii A. Rabiner, Paul Bech, Ali Abbara, David Goldmeier, Alexander N. Comninos, Waljit S. Dhillo

**Affiliations:** 1 Section of Endocrinology and Investigative Medicine, Imperial College London, London, United Kingdom; 2 Invicro LLC, Hammersmith Hospital Campus, London, United Kingdom; 3 Statsconsultancy Ltd, Amersham, United Kingdom; 4 Jane Wadsworth Sexual Function Clinic, St Mary’s Hospital, Imperial College Healthcare NHS Trust, London, United Kingdom; 5 Department of Endocrinology, Imperial College Healthcare NHS Trust, London, United Kingdom

## Abstract

**Importance:**

The human physiological sexual response is crucial for reward, satisfaction, and reproduction. Disruption of the associated neurophysiological pathways predisposes to low sexual desire; the most prevalent psychological form is hypoactive sexual desire disorder (HSDD), which affects 8% of men but currently has no effective pharmacological treatment options. The reproductive neuropeptide kisspeptin offers a putative therapeutic target, owing to emerging understanding of its role in reproductive behavior.

**Objective:**

To determine the physiological, behavioral, neural, and hormonal effects of kisspeptin administration in men with HSDD.

**Design, Setting, and Participants:**

This double-blind, 2-way crossover, placebo-controlled randomized clinical trial was performed at a single academic research center in the UK. Eligible participants were right-handed heterosexual men with HSDD. Physiological, behavioral, functional magnetic resonance imaging (fMRI), and hormonal analyses were used to investigate the clinical and mechanistic effects of kisspeptin administration in response to visual sexual stimuli (short and long video tasks). The trial was conducted between January 11 and September 15, 2021, and data analysis was performed between October and November 2021.

**Interventions:**

Participants attended 2 study visits at least 7 days apart, in balanced random order, for intravenous infusion of kisspeptin-54 (1 nmol/kg/h) for 75 minutes or for administration of a rate-matched placebo.

**Main Outcomes and Measures:**

Changes in (1) brain activity on whole-brain analysis, as determined by fMRI blood oxygen level–dependent activity in response to visual sexual stimuli during kisspeptin administration compared with placebo, (2) physiological sexual arousal (penile tumescence), and (3) behavioral measures of sexual desire and arousal.

**Results:**

Of the 37 men randomized, 32 completed the trial. Participants had a mean (SD) age of 37.9 (8.6) years and a mean (SD) body mass index of 24.9 (5.4). On viewing sexual videos, kisspeptin significantly modulated brain activity in key structures of the sexual-processing network on whole-brain analysis compared with placebo (mean absolute change [Cohen *d*] = 0.81 [95% CI, 0.41-1.21]; *P* =  .003). Furthermore, improvements in several secondary analyses were observed, including significant increases in penile tumescence in response to sexual stimuli (by up to 56% more than placebo; mean difference = 0.28 units [95% CI, 0.04-0.52 units]; *P* = .02) and behavioral measures of sexual desire—most notably, increased happiness about sex (mean difference = 0.63 points [95% CI, 0.10-1.15 points]; *P* = .02).

**Conclusions and Relevance:**

Collectively, this randomized clinical trial provides the first evidence to date showing that kisspeptin administration substantially modulates sexual brain processing in men with HSDD, with associated increases in penile tumescence and behavioral measures of sexual desire and arousal. These data suggest that kisspeptin has potential as the first pharmacological treatment for men with low sexual desire.

**Trial Registration:**

isrctn.org Identifier: ISRCTN17271094

## Key Points

**Question:**

Does kisspeptin administration modulate sexual brain processing in men with low sexual desire due to hypoactive sexual desire disorder (HSDD)?

**Findings:**

In this randomized clinical trial of 32 men with HSDD, kisspeptin administration significantly modulated brain activity in key structures of the sexual-processing network vs placebo and increased sexual behavior and penile tumescence in response to visual sexual stimuli.

**Meaning:**

These data provide early promise of efficacy for the pharmacological use of kisspeptin-based therapeutics as a treatment for men with low sexual desire.

## Teaser

This randomized clinical trial investigates the physiological, behavioral, neural, and hormonal effects of kisspeptin administration in men with hypoactive sexual desire disorder.

## Introduction

The human sexual response is crucial for reward, satisfaction, and reproduction.^1^ Dysregulation and imbalance in the neurophysiological excitatory and inhibitory pathways regulating sexual desire and arousal result in low sexual desire.^2^

One of the most common forms of low sexual desire is hypoactive sexual desire disorder (HSDD), which affects up to 8% of men.^3^ Recent insights suggest that HSDD is triggered by hyperactivation in areas involved in self-referential processing and hypoactivation in brain regions mediating sexual desire.^4^ The resulting shift in attentional focus from sexual stimuli to self-monitoring causes a persistent deficiency of sexual desire with marked distress.^4,5^ Consequently, HSDD has major detrimental effects on quality of life, interpersonal relationships, and fertility.^6,7^ Despite the high clinical burden, there are no licensed pharmacotherapies for men or treatments in development.^8^ Meanwhile, off-label use of agents such as phosphodiesterase 5 inhibitors is ineffective, given that the increased genital response does not primarily target sexual desire.^9,10^ In addition, previous studies highlight that testosterone supplementation in eugonadal men with sexual dysfunction does not improve sexual function.^11^ Thus, novel clinical strategies are much needed.

The neuropeptide kisspeptin is a crucial endogenous activator of the reproductive system,^12,13^ with extensive distribution throughout the rodent^14^ and human^15,16,17^ brain. Emerging evidence from animal models reveals that kisspeptin signaling has key roles in modulating reproductive behavior,^18^ including sexual motivation^19^ and erections.^20^ Moreover, we have previously shown in healthy men that kisspeptin administration enhances limbic activity in response to sexual stimuli, with reductions in sexual aversion.^21^ Combined, these data led us to hypothesize that kisspeptin administration would enhance sexual brain processing and penile tumescence in men with HSDD.

To test our hypothesis, we used physiological, behavioral, functional neuroimaging (functional magnetic resonance imaging [fMRI]), and hormonal analyses to investigate the clinical and mechanistic effects of kisspeptin administration in men with HSDD.

## Methods

This randomized clinical trial followed the Consolidated Standards of Reporting Trials (CONSORT) reporting guideline. The trial protocol is provided in Supplement 1.

### Ethics Approval

This study was approved by the London Riverside Research Ethics Committee in the UK, prospectively registered on the ISRCTN Registry, and performed in accordance with the Declaration of Helsinki.^22^ The trial was conducted between January 11 and September 15, 2021. All participants provided informed written consent before inclusion.

### Participants

Right-handed heterosexual men concerned about and/or distressed by low sexual desire were invited to take part in this trial via advertisements. In a detailed medical screening visit, a diagnosis of HSDD was ascertained (detailed in the eMethods in Supplement 2). Participants completed a battery of psychometric questionnaires (to assist in the diagnosis of HSDD and exclude confounding active depression, anxiety trait, and underlying erectile dysfunction) as well as blood testing. All participants were eugonadal (Table). In addition, data on race and ethnicity were collected at screening through self-reported identification according to the UK Government classification system (Asian, Black, White, mixed or multiple ethnic groups, or other ethnic group).^23^

**Table.** Participant Clinical and Psychometric Characteristics^a^

Characteristic	Treatment sequence^b^		All participants (N = 32)
	Placebo/kisspeptin (n = 16)	Kisspeptin/placebo (n = 16)	
Age, y	36.1 (7.8)	39.7 (9.3)	37.9 (8.6)
Body mass index^c^	24.0 (7.0)	25.9 (3.0)	24.9 (5.4)
Baseline hormone profile (reference range)			
Kisspeptin, pmol/L	31.3 (27.0)	18.1 (7.1)	24.7 (20.6)
Luteinizing hormone (2-12), IU/L	3.3 (1.4)	2.9 (1.3)	3.1 (1.3)
Follicle-stimulating hormone (1.7-8), IU/L	4.2 (3.5)	3.3 (1.6)	3.8 (2.7)
Testosterone (10-30), nmol/L	20.0 (7.0)	16.9 (5.0)	18.5 (6.2)
Sex hormone–binding globulin (15-55), nmol/L	37.1 (12.3)	34.5 (11.8)	35.8 (12.0)
Thyroid-stimulating hormone (0.3-4.2), mIU/L	1.4 (0.4)	1.5 (0.7)	1.4 (0.6)
Free T_4_ (9-23), pmol/L	12.0 (1.0)	11.3 (2.2)	11.6 (1.7)
Duration of current relationship, y	6.9 (6.0)	8.7 (8.9)	7.8 (7.5)
Overall satisfaction with partner^d^	3.3 (1.2)	3.4 (0.9)	3.3 (1.0)
No. of sexual partners in past year	1.1 (0.6)	0.9 (0.5)	1.0 (0.6)
Frequency of sexual intercourse per month	1.6 (1.1)	3.1 (3.3)	2.3 (2.5)
Hours viewing pornographic material per week	0.7 (1.1)	0.8 (1.3)	0.8 (1.2)
Psychometric assessment			
Behavioral Inhibition System Scale^e^	20.9 (3.3)	20.4 (2.4)	20.6 (2.8)
Behavioral Activation and System Scale^f^			
Drive	11.4 (2.2)	10.9 (2.7)	11.2 (2.5)
Fun	12.2 (2.4)	11.6 (1.7)	11.9 (2.1)
Reward	16.8 (2.1)	16.4 (2.2)	16.6 (2.2)
Generalized Anxiety Disorder Assessment-7^g^	2.1 (1.6)	1.8 (3.3)	1.9 (2.6)
International Index of Erectile Function^h^			
Erectile function	26.5 (8.2)	27.1 (9.0)	26.8 (4.5)
Orgasmic function	6.6 (2.6)	5.9 (3.2)	6.2 (2.9)
Sexual desire	3.9 (1.2)	4.5 (2.4)	4.2 (1.9)
Intercourse satisfaction	6.9 (2.7)	6.2 (3.9)	6.5 (3.3)
Overall satisfaction	4.3 (2.1)	4.6 (1.6)	4.4 (1.9)
Patient Health Questionnaire-9^i^	2.6 (2.7)	2.3 (2.3)	2.5 (2.5)
Sexual Desire Inventory^j^			
Dyadic	21.7 (7.2)	25.8 (10.8)	23.7 (9.3)
Solitary	9.4 (4.7)	7.2 (3.6)	8.3 (4.2)
Total	38.6 (12.8)	40.4 (15.4)	39.5 (14.0)
Sexual Concerns Inventory–Male^k^			
Desire distress domain	3.3 (0.9)	3.2 (0.9)	3.3 (0.9)
Total	29.3 (7.6)	29.0 (8.4)	29.1 (7.9)
Subjective Happiness Scale^l^	17.5 (5.6)	18.1 (4.3)	4.4 (1.2)
Sexual Quality of Life–Male^m^	31.5 (14.8)	32.8 (11.5)	32.2 (13.0)
State-Trait Anxiety Inventory Trait^n^	41.8 (10.1)	38.6 (6.8)	40.2 (8.6)
Satisfaction With Life Scale^o^	23.4 (6.7)	22.0 (5.3)	22.7 (6.0)

Abbreviation: T_4_, levorotatory thyroxine.

^a^ Data are presented as the mean (SD).

^b^ Sixteen participants received placebo at the first study visit and kisspeptin at the second study visit; 16 participants received kisspeptin at the first study visit and placebo at the second study visit.

^c^ Calculated as weight in kilograms divided by height in meters squared.

^d^ Scored on a scale from 0 to 5, where 0 indicates very unsatisfied and 5 very satisfied.

^e^ Sensitivity to anticipation of punishment (score range, 1-28; higher scores indicate greater motivation to avoid aversive outcomes).

^f^ Sensitivity to desired goals, fun, and reward (score ranges: drive, 1-16; fun, 1-16; and reward, 1-20; higher scores indicate greater motivation to approach goal-orientated outcomes).

^g^ Anxiety screening (score range, 0-21; scores ≥5 indicate anxiety).

^h^ Domains of male sexual function (score ranges: erectile function, 0-30; orgasmic function, 0-10; sexual desire, 0-10; intercourse satisfaction, 0-15; and overall satisfaction, 0-10; higher scores indicate better sexual function).

^i^ Depression screening (score range, 0-27; scores ≥5 indicate depression).

^j^ Dyadic (ie, with a partner) and solitary sexual desire (score ranges: dyadic domain, 0-62; solitary domain, 0-23; and total, 0-109; higher scores indicate higher sexual desire).

^k^ Sex-related distress in men (score ranges: desire distress, 0-4; and total, 0-48; higher scores indicate higher sex-related distress).

^l^ Overall subjective happiness (score range, 1-7; higher scores indicate greater happiness).

^m^ Effect of sexual dysfunction on quality of life (score range, 11-66; higher scores indicate better sexual quality of life).

^n^ Trait anxiety (score range, 20-80; higher scores indicate higher levels of anxiety).

^o^ Satisfaction with life as a whole (scores range, 5-35; higher scores indicate higher satisfaction with life).

### Study Design

**Figure 1.**
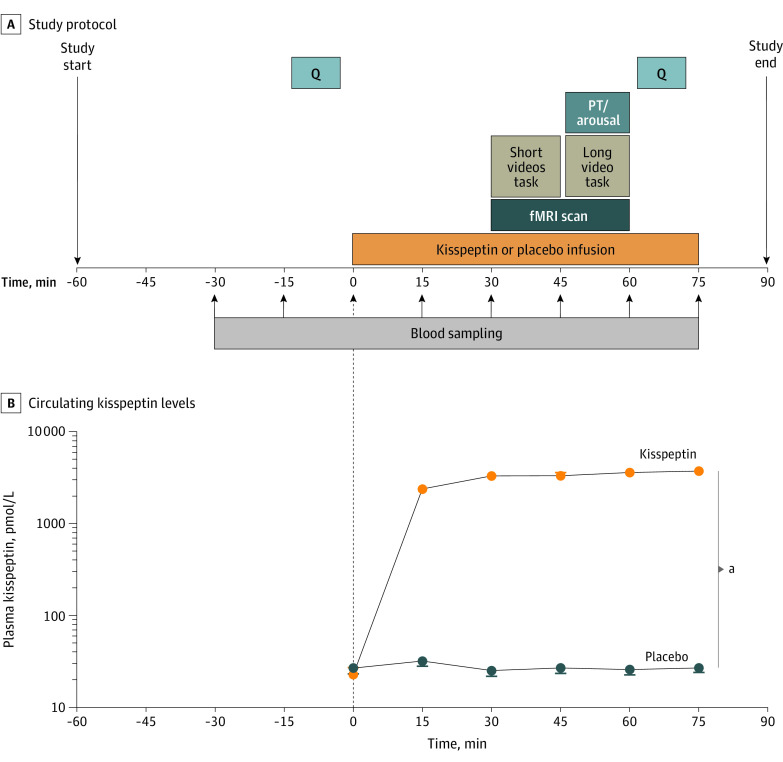
Experimental Protocol and Effects of Kisspeptin on Circulating Kisspeptin Levels A, Thirty-two men with hypoactive sexual desire disorder completed this double-blind, 2-way crossover, placebo-controlled randomized clinical trial. They attended 2 study visits at least 7 days apart, in balanced random order. One visit was for intravenous administration of kisspeptin-54 (1 nmol/kg/h), and the other was for an equivalent volume of placebo for 75 minutes. At each visit, functional magnetic resonance imaging (fMRI) was performed, with participants completing short and long sexual video tasks. The short videos task consisted of participants watching 20-second sexual videos (depicting heterosexual couples engaging in sexual intercourse), alternating with control nonsexual segments (involving 1 woman and 1 man exercising). The long video task consisted of participants watching a continuous 8-minute video depicting a heterosexual couple engaging in sexual activities, during which objective sexual arousal data (penile tumescence [PT]) and the subjective level of arousal were recorded. Participants completed psychometric questionnaires (Q) before and during kisspeptin and placebo administration. B, Kisspeptin administration resulted in increased circulating kisspeptin levels, reaching a plateau at 30 minutes. Therefore, circulating kisspeptin levels were stable during fMRI and intrainfusion psychometric assessments. Data depict the mean (SEM). ^a^*P* < .001 (2-way analysis of variance).

In this double-blind, 2-way crossover, placebo-controlled randomized clinical trial, participants completed 2 study visits each (kisspeptin and placebo) at least 7 days apart (Figure 1A). Detailed methods are provided in the eMethods in Supplement 2. Participants had 2 intravenous cannulas sited for blood sampling and for kisspeptin or placebo administration. Blood sampling for hormonal measurements took place at 15-minute intervals from 30 minutes before commencement of the kisspeptin or placebo infusion to the end of the 75-minute infusion. At 0 minutes, a 75-minute infusion of kisspeptin-54 (1 nmol/kg/h) or placebo (using rate-matched 4% succinylated gelatin solution [Gelofusine; B. Braun]) commenced. Participants completed psychometric questionnaires before and toward the end of kisspeptin or placebo administration (eMethods in Supplement 2).

Between 30 and 60 minutes of the kisspeptin or placebo infusion, fMRI scanning was performed with participants completing 2 tasks as described next (and in the eMethods in Supplement 2).

#### Short Videos Task

In the short videos task, participants watched 20-second segments of sexual content (depicting heterosexual couples engaging in sexual intercourse) alternating with control nonsexual segments (involving 1 woman and 1 man exercising) for a total of 12 minutes.

#### Long Video Task

In the long video task, participants watched a continuous 8-minute video depicting a heterosexual couple engaging in sexual activities, during which objective arousal (measured using penile tumescence) and subjective arousal (using a magnetic resonance–compatible scroll wheel) were recorded continuously and used to interrogate the fMRI brain data (eMethods in Supplement 2).

### Outcome Measures

The primary outcome was change in brain activity on whole-brain analysis as determined by fMRI blood oxygen level–dependent (BOLD) activity in response to visual sexual stimuli during kisspeptin administration compared with placebo. Secondary outcomes were as follows: changes in behavioral measures of sexual desire and arousal, general mood, anxiety, and nonsexual attention; correlation analyses between brain activity in a priori–defined regions of interest (ROIs) and psychometric scores; changes in physiological arousal as measured by penile tumescence; and changes in hormone levels during kisspeptin administration compared with placebo. Safety assessments included adverse events, blood pressure, and heart rate recordings.

### Sample Size

To our knowledge, this is the first fMRI study in men with HSDD, so no equivalent data were available to guide sample size calculations. However, our previous work demonstrates that kisspeptin enhances task-based brain activation (measured by the percentage of fMRI BOLD signal change) by a mean (SD) of 0.74% (0.38%) in healthy men compared with placebo.^21^ We anticipated a similar response in men with HSDD. Using these data, with an α of .05, power of 0.8, and assuming correlation between means of 0.40, a power calculation was performed, resulting in a sample size of 31. Our final sample size of 32 participants also compares favorably with our previous studies examining the effects of kisspeptin on fMRI brain activity in healthy men^21,24,25,26^ and women with HSDD^27^ as well as empirically derived estimates of optimal sample sizes in fMRI studies.^28,29^ To allow for dropouts and exclusions, 37 participants were recruited.

### Statistical Analysis

Statistical analyses (including power calculation) were performed between October and November 2021 in collaboration with a statistician (P.A.B.) using Prism, version 9.3 (GraphPad Software). Hormone, psychometric, and physiological data were normally distributed by Kolmogorov testing. Penile tumescence and hormone data were analyzed using 2-way analysis of variance with the Bonferroni multiple-comparison test. The maximal difference in penile tumescence between kisspeptin and placebo was analyzed using paired 2-tailed *t* tests. Differences between baseline and change in psychometric scores during kisspeptin administration compared with placebo were analyzed using paired 2-tailed *t* tests. Pearson correlation was used to assess correlations between brain activity in a priori–defined ROIs with behavioral measures, in keeping with our and others’ previous work.^21,24,25,27,30,31^ To adjust for the number of analyses, a reduced α threshold from standard *P* < .05 to *P* < .01 identified statistical significance in the correlation analyses, in line with previous work.^21^

## Results

### Participant Characteristics

Of the 37 men randomized, 32 completed this trial (eFigure 1 in Supplement 2). Participants had a mean (SD) age of 37.9 (8.6) years and a mean (SD) BMI of 24.9 (5.4). With regard to race and ethnicity, 6 participants (19%) were Asian, 4 (13%) were Black, and 22 (68%) were White. Baseline characteristics are provided in the Table.

### Effects of Kisspeptin on Circulating Reproductive Hormone Levels

Before kisspeptin or placebo administration, kisspeptin, gonadotropin, testosterone, and cortisol levels were equivalent between visits (eTable 1 in Supplement 2). As expected, intravenous kisspeptin significantly increased circulating kisspeptin levels (mean difference = 2705 pmol/L [95% CI, 2560-2851 pmol/L]; *P* < .001), reaching steady state from 30 to 75 minutes of the kisspeptin infusion (Figure 1B). Therefore, circulating kisspeptin levels were stable during the fMRI and intrainfusion psychometric assessments. In parallel, kisspeptin significantly increased luteinizing hormone (LH) and follicle-stimulating hormone (FSH) to similar levels reported using this administration protocol,^21,24,25,26,27,32^ confirming bioactivity (eFigure 2A and B in Supplement 2). No changes in downstream testosterone or cortisol (eFigure 2C and D in Supplement 2) were observed during the 75-minute study period, in keeping with previous work.^21,24,25,26,32^

### Effects of Kisspeptin on Brain Activity During the Short Sexual Videos Task

In the sexual compared with control exercise videos contrast (primary outcome), kisspeptin significantly modulated activity in key sexual-processing brain structures compared with placebo (mean absolute change [Cohen *d*] = 0.81 [95% CI, 0.41-1.21]; *P* = .003).

**Figure 2.**
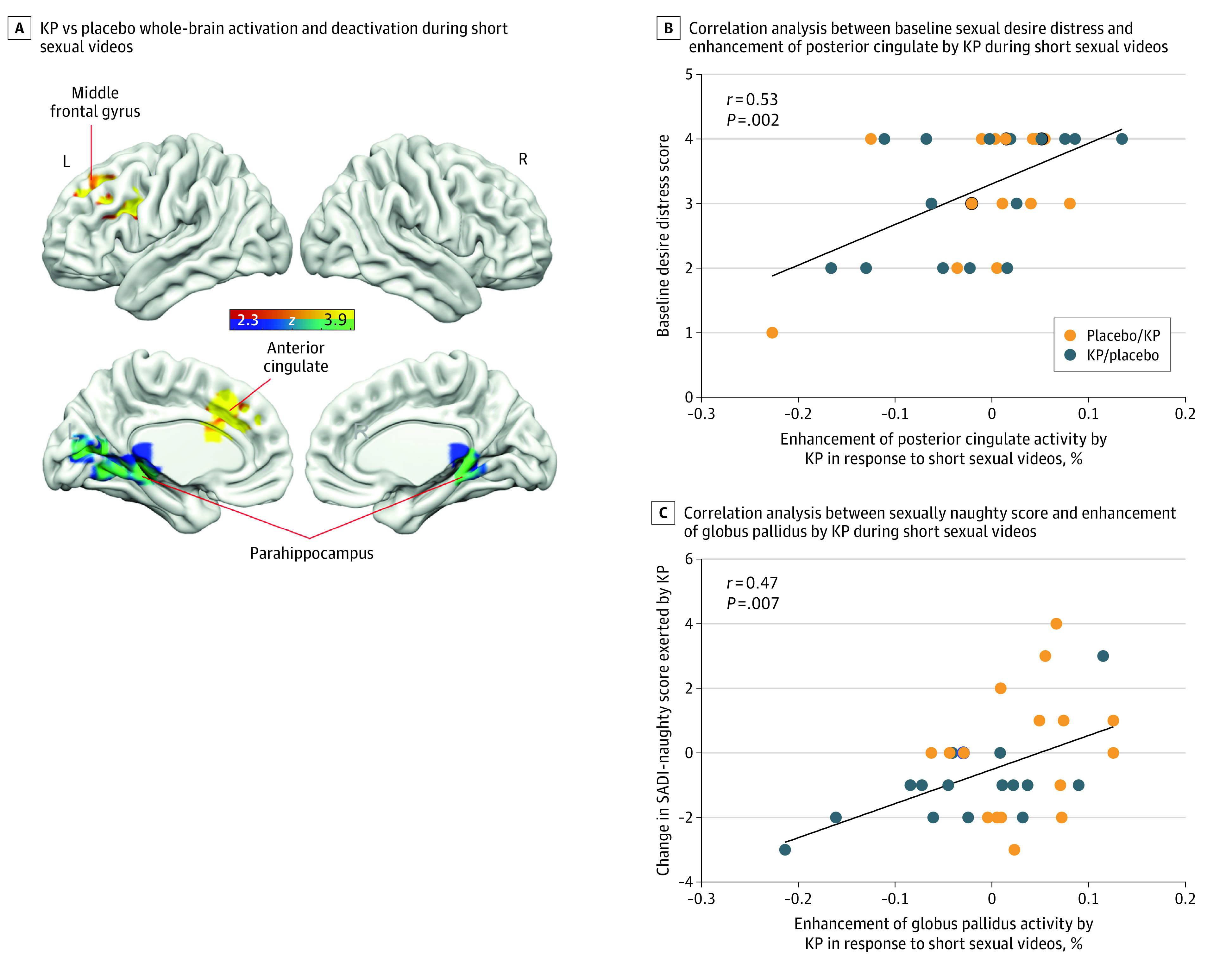
Effects of Kisspeptin (KP) on Sexual Brain Activity and Behavior During the Short Sexual Videos Task A, Whole-brain analysis showing increased activity in the left anterior cingulate and left middle frontal gyrus and decreased activity in the bilateral parahippocampus by KP administration in response to short sexual videos. Red and yellow areas show relative activation to sexual vs exercise videos (control) during KP administration compared with placebo. Blue and green areas show relative deactivation to sexual vs exercise videos during KP administration compared with placebo. Clusters are corrected for multiple comparisons (*z* = 2.3; *P* < .05 for all comparisons; N = 32). L indicates left; R, right. B, Correlation analysis demonstrating that participants with higher baseline sexual desire distress scores showed greater KP-enhanced brain activity in the posterior cingulate cortex in response to short sexual videos compared with exercise videos. In B and C, placebo/KP depicts the 16 participants who received placebo at the first study visit and KP at the second study visit; KP/placebo depicts the 16 participants who received KP at the first study visit and placebo at the second study visit. C, Correlation analysis demonstrating that the more KP enhanced globus pallidus activity, the more sexually “naughty” the participants felt in response to short sexual videos compared with exercise videos.

Specifically, kisspeptin significantly enhanced activation in the left middle frontal gyrus (MFG) (*d* = 0.82 [95% CI, 0.42-1.22]; *P* = .001) and left anterior cingulate cortex (ACC) (*d* = 0.77 [95% CI, 0.37-1.16]; *P* = .001) (Figure 2A; eTables 2 and 3 in Supplement 2). In the same contrast, relative deactivation was observed in the bilateral parahippocampus (*d* = −0.84 [95% CI, −1.24 to −0.43]; *P* = .007) (Figure 2A; eTables 2 and 3 in Supplement 2).

Correlating brain activity in the anatomical ROIs with behavioral measures revealed that participants with higher baseline sexual desire distress scores showed greater kisspeptin-enhanced brain activity in the posterior cingulate cortex (PCC) on viewing short sexual videos (using Sexual Concerns Inventory–Male desire distress score, *r* = 0.53 [95% CI, 0.23-0.74]; *P* = .002) (Figure 2B). In addition, the degree of kisspeptin-enhanced activity in the globus pallidus on viewing short sexual videos positively correlated with how sexually “naughty” participants felt (using Sexual Arousal and Desire Inventory [SADI]–naughty scores; *r* = 0.47 [95% CI, 0.14-0.70]; *P* = .007) (Figure 2C).

### Effects of Kisspeptin on Penile Tumescence During the Long Sexual Video Task

**Figure 3.**
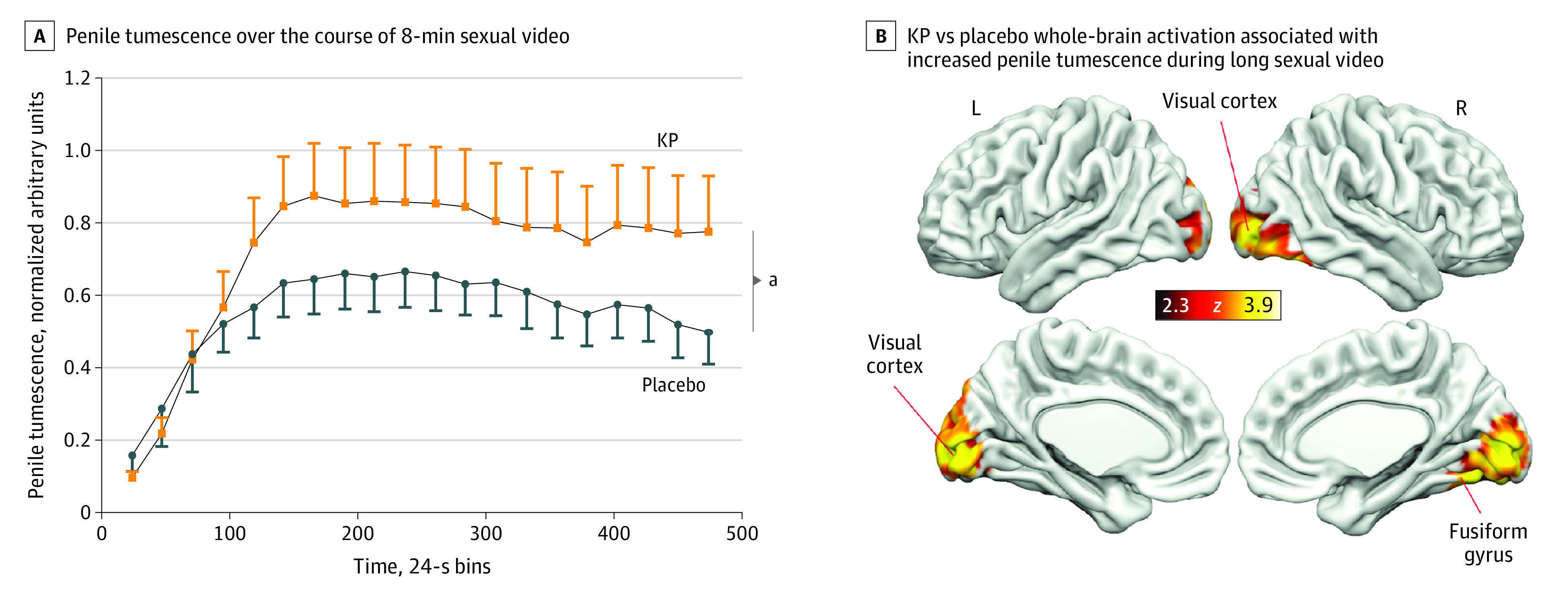
Effects of Kisspeptin (KP) on Physiological Measures of Sexual Arousal (Penile Tumescence) and Sexual Brain Activity During the Long Sexual Video Task A, Kisspeptin administration resulted in a significant increase in penile tumescence over the course of an 8-minute sexual video compared with placebo. The maximal response occurred at 8 minutes, with KP administration increasing penile tumescence by 56% more than placebo while participants watched the long sexual video. Error bars show the SEM. B, Whole-brain analysis showing increased bilateral visual cortex and right fusiform gyrus activity by KP administration in response to the long sexual video as penile tumescence increased. Red and yellow areas show activation as penile tumescence increased during KP administration compared with placebo. Clusters are corrected for multiple comparisons (*z* = 2.3; *P* < .05 for all comparisons; n = 30). L indicates left; R, right. ^a^*P* = .002 (n = 30).

In response to the 8-minute sexual video, kisspeptin significantly increased penile tumescence compared with placebo (*F*_19,551_ = 2.27; *P* = .002) (Figure 3A). Kisspeptin’s proerectile effect was most marked by the end of the 8-minute task, with kisspeptin augmenting penile tumescence by up to 56% more than placebo while participants watched the long sexual video (mean difference = 0.28 units [95% CI, 0.04-0.52 units]; *P* = .02) (Figure 3A).

### Effects of Kisspeptin on Brain Activity During the Long Sexual Video Task

#### Penile Tumescence

Using objective arousal data from the penile tumescence device as a regressor (to identify brain regions where activity was related to tumescence), kisspeptin increased activity in the right fusiform gyrus (*d* = 0.27 [95% CI, 0.10-0.63]; *P* < .001) and bilateral visual cortex (*d* = 0.50 [95% CI, 0.12-0.88]; *P* < .001) compared with placebo. This observation indicated that activity in these areas was more related to increasing penile tumescence during kisspeptin administration than placebo (Figure 3B; eTables 2 and 3 in Supplement 2).

#### Sexual Arousal

**Figure 4.**
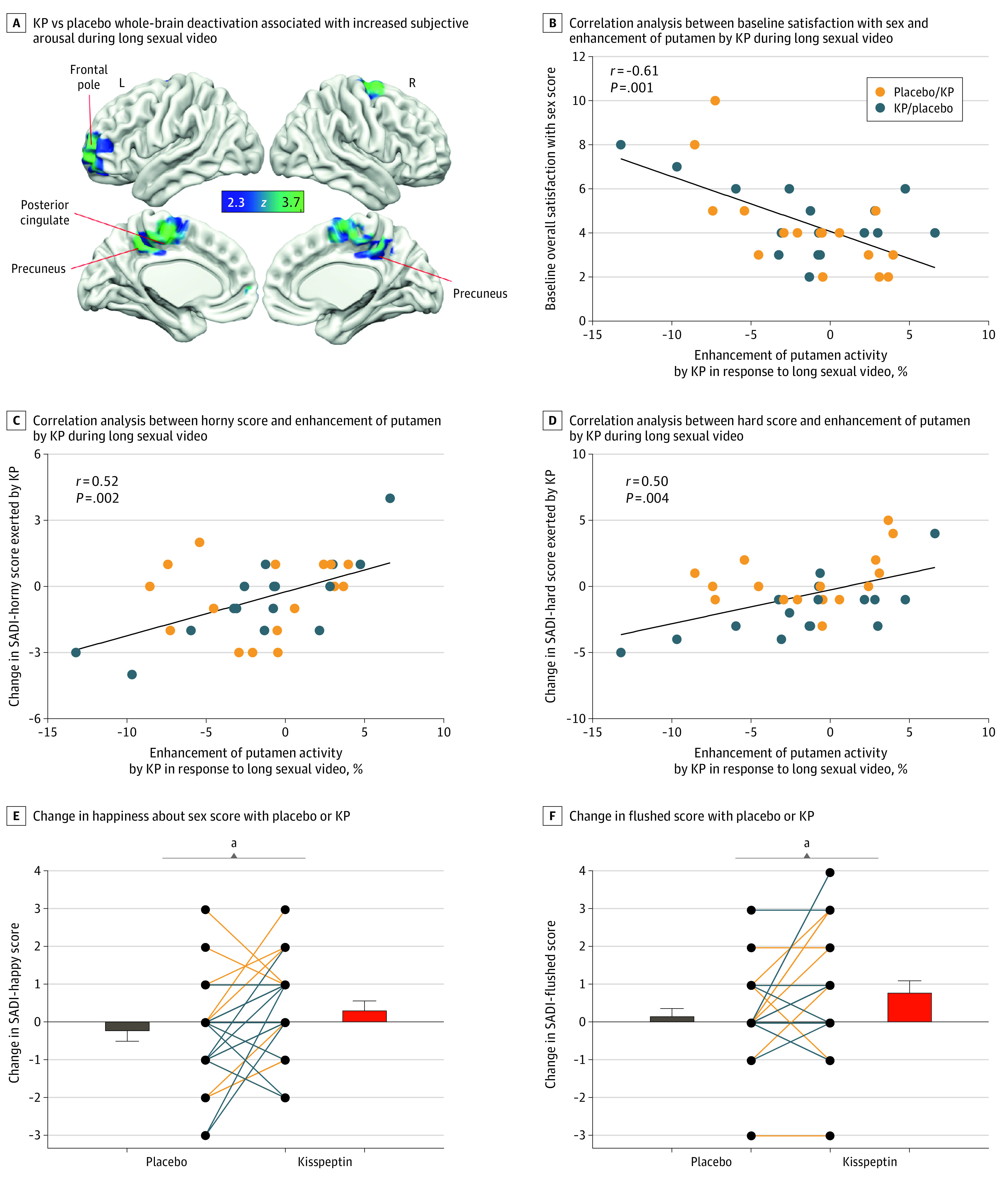
Effects of Kisspeptin (KP) on Sexual Brain Activity During the Long Sexual Video Task as Subjective Arousal Increased and Behavioral Parameters of Sexual Desire and Arousal A, Whole-brain analysis showing decreased activity in the left frontal pole, right posterior cingulate cortex, and bilateral precuneus by KP administration in response to the long sexual video as subjective arousal increased. Blue and green areas show deactivation as subjective arousal increased during KP administration compared with placebo. Clusters are corrected for multiple comparisons (*z* = 2.3; *P* < .05 for all comparisons; N = 32). L indicates left; R, right. B, Correlation analysis. Participants with lower baseline satisfaction with sex showed greater KP-enhanced brain activity in the putamen in response to the long sexual video. B through F, Placebo/KP depicts the 16 participants who received placebo at the first study visit and KP at the second; KP/placebo depicts the 16 participants who received KP at the first study visit and placebo at the second. C, The more KP enhanced putamen activity, the more “horny” the participants felt in response to the long sexual video. D, The more KP enhanced putamen activity, the more “hard” the participants felt in response to the long sexual videos. E and F, Score changes for each participant show that kisspeptin administration significantly increased participant-reported happiness about sex (E) and flushing (F) compared with placebo. ^a^*P* = .02 (paired 2-sided *t* test).

Using subjective arousal as a regressor (to identify brain regions where activity was related to subjective arousal), kisspeptin decreased activity in the left frontal pole (*d* = −0.46 [95% CI, −0.82 to −0.09]; *P* = .04), right PCC (*d* = −0.59 [95% CI, −0.96 to −0.21]; *P* = .001), and bilateral precuneus (*d* = −0.42 [95% CI, −0.78 to −0.06]; *P* = .001). This observation indicated that activity in these areas was more related to increasing subjective arousal during kisspeptin administration than placebo (Figure 4A; eTables 2 and 3 in Supplement 2).

Correlating brain activity in the anatomical ROIs with behavioral measures, participants who reported a lower baseline overall satisfaction with sex showed greater kisspeptin-enhanced activity in the putamen (using the International Index of Erectile Function–Intercourse Satisfaction score, *r* = −0.61 [95% CI, −0.79 to −0.33]; *P* < .001) (Figure 4B). In addition, we observed that kisspeptin’s enhanced activation of the putamen related to increased measures of sexual desire and arousal (SADI-horny score: *r* = 0.52 [95% CI, 0.21-0.74]; *P* = .002; and SADI-hard score: *r* = 0.50 [95% CI, 0.18-0.72]; *P* = .004) (Figure 4C and D).

### Effects of Kisspeptin on Behavioral Measures of Sexual Desire and Arousal

Kisspeptin administration increased participant-reported happiness about sex compared with placebo (using SADI-happy score, mean difference = 0.63 points [95% CI, 0.10-1.15]; *P* = .02) (Figure 4E). Kisspeptin also increased participant-reported flushing (using SADI-flushed score, mean difference = 0.63 points [95% CI, 0.11-1.14]; *P* = .02) (Figure 4F). There was no effect at a domain level (eFigure 3 in Supplement 2).

### Effects of Kisspeptin on General Mood, Anxiety, and Nonsexual Attention

Patients with HSDD are known to have increased risk of abnormal mood and anxiety symptoms.^5^ No difference in general mood, anxiety, and nonsexual attention was observed (eFigure 4 in Supplement 2).

### Safety

Kisspeptin was well tolerated, with no side effects or adverse events reported (eTable 4 in Supplement 2). In addition, kisspeptin had no significant clinical effects on blood pressure or heart rate (eFigure 5 in Supplement 2), as previously reported.^33^

## Discussion

This randomized clinical trial provides the first clinical evidence, to our knowledge, showing that kisspeptin administration in men with low sexual desire modulates sexual brain processing, enhances penile tumescence in response to sexual stimuli (by up to 56% more than placebo), and improves behavioral measures of sexual desire and arousal.

Notably, in this study, kisspeptin increased happiness about sex as well as flushing. Flushing is a common feature encountered by 25% of men during sexual arousal.^34^ Mechanistic insight for these observations may be provided by kisspeptin’s effects on brain activity.

In the short videos task, kisspeptin modulated activity in 3 distinct and relevant brain regions (left MFG, left ACC, and bilateral parahippocampus). The left MFG, a region expressing kisspeptin receptors,^14^ is an important component of the executive attention network.^35^ Therefore, kisspeptin’s enhancement of this area is pertinent given that the more attention a person allocates to sexual stimuli, the more sexual arousal is experienced,^36^ whereas individuals with sexual dysfunction typically display less sustained attention to sexual stimuli.^37^ Collectively, left MFG activation by kisspeptin may enable men with HSDD to increase attentional capture of sexual cues, providing a neural mechanism facilitating arousal.

In addition, kisspeptin enhanced left ACC activity. This brain region also expresses kisspeptin receptors^15^ and is involved in the motivational and autonomic components of sexual desire and arousal.^38^ Previous neuroimaging studies in healthy men identified ACC activation to be highly correlated with the intensity of perceived arousal and the magnitude of penile tumescence.^38,39^ Notably, in a noninterventional positron emission tomography study, activation of the left ACC was observed in healthy men in response to sexual stimuli, whereas activity was unchanged in men with HSDD.^40^ Therefore, in the context of these established ACC roles, kisspeptin’s enhancement in the current study may heighten the motivation for sexual activity in men with HSDD, providing further mechanistic insight.

In the short sexual videos task, we observed deactivation of the bilateral parahippocampus, which also expresses kisspeptin receptors.^15^ This finding is consistent with parahippocampal deactivation in a positron emission tomography study of healthy men who were presented with sexual stimuli.^38^ Functionally, the parahippocampus is implicated in introspective self-monitoring^41^; this is relevant given that in HSDD, a shift in attentional focus from sexual stimuli to self-monitoring inhibits normal sexual function.^4^ In line with these findings, kisspeptin decreased parahippocampal activity in the current study, which may serve to downregulate introspective processes, thereby promoting arousal.

During the long video task, we observed that kisspeptin elicited a marked increase in penile tumescence, providing evidence for kisspeptin’s proerectile effects in humans. Regarding the neural pathways involved, kisspeptin increased activity in 2 key visual brain regions (right fusiform gyrus and bilateral visual cortex). This finding is consistent with a previous meta-analysis of fMRI studies in healthy men, which reported that exposure to sexual stimuli consistently induced marked fusiform gyrus activation^42^ to recognize a stimulus as sexual.^43,44^ Similarly, visual cortex activation is frequently observed in men in response to sexual stimuli.^45^ Collectively, kisspeptin’s enhancement of the fusiform gyrus and visual cortex in the present study may augment engagement and sexual attention to high-level visual features of sexual cues to enhance penile tumescence.

Regarding the neural pathways involved in subjective arousal, kisspeptin decreased activity in 3 distinct brain regions (right PCC, bilateral precuneus, and left frontal pole) during the long video task. Kisspeptin’s deactivation of the right PCC and bilateral precuneus is concordant with a previous fMRI study in healthy men, which reported similar deactivations in response to sexual stimuli.^46^ Notably, both regions are implicated in self-referential functions.^47^ Indeed, an fMRI meta-analysis in women with HSDD revealed hyperactivation in both regions, implying that a related increase in self-focus may interfere with normal sexual function.^4^ In our study, kisspeptin decreased activity in these regions; this would therefore promote attention to sexual stimuli, while reducing self-focus that may otherwise disrupt sexual function in HSDD.

Kisspeptin administration during the long video task also deactivated the left frontal pole, another region that expresses kisspeptin receptors^14^ and is implicated in self-control.^48^ Frontal pole activity is tightly coupled to sexual arousal, such that decreased activity is associated with higher arousal.^48^ In healthy men, escalating arousal during genital stimulation causes a decrease in frontal pole activity, whereas activation becomes most prominent after stimulation ceases.^49^ Thus, frontal pole deactivation enables arousal by dissolving normal inhibitory boundaries.^48^ Therefore, deactivation of this area by kisspeptin provides further insight for the mechanism causing increased arousal in our study.

Furthermore, we observed significant correlations between kisspeptin-enhanced brain activity and important baseline behavioral parameters. In men reporting higher baseline sex-related distress, greater kisspeptin enhancement was observed in the PCC, a key reward and motivation structure^50^ expressing kisspeptin receptors,^15^ on viewing short sexual videos. A similar relationship was demonstrated between kisspeptin’s enhancement of putamen activity in response to the long sexual video, which was more pronounced in men reporting lower baseline satisfaction with sex. The putamen is a dopamine-rich region expressing kisspeptin receptors^15^ involved in reward and motivation.^51^ Our behavioral findings suggest that kisspeptin’s enhancement of these structures may provide a functional mechanism for enhancing reward and motivation in response to sexual stimuli in individuals with a lower sexual quality of life.

In addition to baseline behavioral parameters, we observed significant correlations between kisspeptin-enhanced brain activity and behavioral measures of sexual function in the current moment. The more kisspeptin enhanced globus pallidus activity during short sexual videos, the more sexually “naughty” the participants felt; this finding is relevant given that the globus pallidus expresses kisspeptin receptors^15^ and is implicated in sexual arousal.^42^ In addition, the more kisspeptin enhanced activity in the putamen in response to the long sexual video, the more “horny” and “hard” the participants felt. This finding is pertinent given the established relationship between putamen activity and the degree of penile erection,^52^ providing further mechanistic insight for kisspeptin’s proerectile effect. Collectively, these findings provide key behavioral and functional relevance for kisspeptin’s enhancement of sexual brain activity by serving to strengthen feelings of sexual desire and arousal in men with low sexual desire.

Mechanistically, we administered peripheral kisspeptin-54, which can activate gonadotropin-releasing hormone neuron dendritic terminals before the blood-brain barrier^18^ as well as cross the blood-brain barrier to directly access deeper brain structures expressing kisspeptin receptors.^21^ Indeed, in the present study, kisspeptin modulated brain regions matching kisspeptin receptor expression in rodents^14^ and humans,^15,16,17^ suggesting direct actions of kisspeptin on its receptor as well as brain regions not known to express kisspeptin receptors, indicating additional indirect mechanisms. Animal models demonstrate that kisspeptin also interacts with neurosteroids^53^ and other neurotransmitter systems, including serotonin, dopamine, γ-aminobutyric acid, and nitric oxide.^18^ Moreover, we recently demonstrated through proton magnetic resonance spectroscopy that kisspeptin decreases γ-aminobutyric acid levels in the human limbic system.^32^ Therefore, the effects seen in the present study likely indicate direct actions of kisspeptin on its receptor as well as interactions with neurosteroids and other neurotransmitter systems, with kisspeptin serving as the primary conductor. Of note, kisspeptin administration resulted in significant increases in circulating LH and FSH in this study, as expected. However, it is unlikely that the physiological, behavioral, and neural effects observed are due to these LH and FSH changes, as neither is known to have a direct role in sexual behavior in humans. Moreover, rodent data reveal that several of kisspeptin’s effects on sexual behavior are independent of downstream hormones (including gonadotropin-releasing hormone, LH, or testosterone).^20,54,55^

The promise of efficacy identified by our data raises several directions for future clinical investigation. Kisspeptin can easily and safely be administered via several routes, including by subcutaneous^56,57^ and intranasal delivery.^58^ These delivery routes now warrant evaluation in patients with HSDD, as they are advantageous compared to the intravenous route utilized in the current study. Given the significant stimulatory effect of kisspeptin administration on penile tumescence in this study and its proerectile effect in rodents,^20^ future studies should seek to examine kisspeptin administration in patients with erectile dysfunction. Furthermore, preclinical animal models identify that kisspeptin signaling can modulate mood and emotions^59^; therefore, studies of kisspeptin administration in patients with psychological disorders may prove fruitful (with potentially different durations and follow-up). In view of the substantial increases in sexual function and the availability of kisspeptin antagonists,^20^ future studies could also examine blocking kisspeptin signaling and its potential to restrain sexual desire in certain situations of hypersexuality.

### Strengths and Limitations

This study benefited from several strengths. We used fMRI as an established method to detect arousal by mapping activated brain areas^1^ (eFigures 6-8 in Supplement 2), with participants completing 2 fMRI tasks (short and long sexual videos) designed to induce different sexual response aspects. Study visits were randomized, with participants and data analysts blinded to the intervention administered. All participants interacted with the same male doctor throughout (E.G.M.), thereby reducing bias associated with investigator sex.^60^ Studies commenced in the morning to control for circadian hormonal changes, and both the fMRI tasks and questionnaires were completed prior to any downstream increases in circulating testosterone. Moreover, it is unlikely that free testosterone would have been substantially altered during this time course, given that sex hormone–binding globulin levels do not significantly change during intravenous kisspeptin infusion in men.^61^ Kisspeptin had no effect on circulating cortisol levels, which is pertinent given that anxiety frequently coexists with HSDD.^5^

Regarding limitations, the population of this randomized clinical trial was limited to right-handed heterosexual men to standardize the sexual stimuli used during the fMRI tasks. Whether our findings are generalizable to a wider population remains to be determined in broader cohorts (for instance, including different sexual orientations, broader age groups, and left-handed patients). Moreover, variations in subjective arousal from the sexual stimuli may have been encountered; to mitigate this, an independent focus group was convened to select the most arousing videos. The short videos did not include audio. However, it is established that men generally respond to visual sexual stimuli, whereas women require the addition of auditory and olfactory cues for arousal.^62^ In addition, we may have missed more subtle behavioral effects that may have emerged if participants were in more comfortable surroundings (like their home).

## Conclusions

This randomized clinical trial provides the first clinical evidence to date showing that kisspeptin administration in men with low sexual desire (HSDD) modulates sexual brain activity as well as markedly increases penile tumescence in response to sexual stimuli (by up to 56% more than placebo) and associated behavioral measures of sexual desire and arousal. Taken together, our data suggest that pharmacological use of kisspeptin-based therapeutics may offer the first safe and much-needed clinical strategy for men with HSDD and low sexual desire more broadly.

## Notes

Supplement 1.
Trial Protocol

Supplement 2.
eMethods.

eReferences
**eFigure 1.** Participant Recruitment and Flow Summary **eFigure 2.** Effects of Kisspeptin Administration on Hormone Levels **eFigure 3.** Effects of Kisspeptin Administration on Sexual Arousal and Desire Inventory Scores **eFigure 4.** Effects of Kisspeptin Administration on Positive and Negative Mood, Anxiety, and Nonsexual Attention **eFigure 5.** Effects of Kisspeptin Administration on Blood Pressure and Heart Rate Measurements **eFigure 6.** Group Mean Analysis for Main Effects of the Short Videos Task in All Participants and Both Treatments (Kisspeptin and Placebo) Demonstrating Effective Task Design **eFigure 7.** Group Mean Analysis for Brain Areas Associated With Increasing Penile Tumescence During the Long Video Task in All Participants and Both Treatments (Kisspeptin and Placebo) Demonstrating Effective Task Design **eFigure 8.** Group Mean Analysis of Brain Areas Associated With Increasing Subjective Arousal During the Long Video Task in All Participants and Both Treatments (Kisspeptin and Placebo) Demonstrating Effective Task Design **eTable 1.** Participant Baseline Clinical and Psychometric Characteristics at the Beginning of the Kisspeptin and Placebo Visits **eTable 2.** Clusters With Enhanced Activation or Deactivation by Kisspeptin on Whole-Brain Analysis **eTable 3.** Effect Sizes for Brain Regions With Significantly Enhanced Activation or Deactivation by Kisspeptin Administration **eTable 4.** Safety Outcomes

Supplement 3.
Data Sharing Statement
