# Supplementary material for: Effects of Kisspeptin on Sexual Brain Processing and Penile Tumescence in Men With Hypoactive Sexual Desire Disorder: A Randomized Clinical Trial
Source: JAMA Netw Open. 2023 Feb 3;6(2):e2254313. doi: 10.1001/jamanetworkopen.2022.54313 (PMC9898824; doi:10.1001/jamanetworkopen.2022.54313)
Supplement: Supplement 1. — Trial Protocol [file jamanetwopen-e2254313-s001.pdf]

Department of Investigative Medicine
Imperial College London

6th Floor Commonwealth Building
Imperial College London at
Hammersmith Campus
Du Cane Road, London W12 ONN, UK
Tel: +44 (0) 020 7594 2489
Fax: +44 (0)20 8383 8320

Study Protocol: Physiological Studies to Investigate the Effects of Kisspeptin on Human Brain Processing.

Background

Psychosexual dysfunction affects 1 in 3 people (1) with profound consequences for individuals and their partners, impairing quality of life and interpersonal relationships as well as reducing chances for successful conception (2). Psychosexual dysfunction is caused by problems with sexual functioning due to a psychological rather than a physical condition. It involves several disorders including impotence and reduced sexual desire. In the UK the cost of treating psychosexual disorders with medical and psychological therapy is estimated at £32 million/year (3). Current medical treatments are limited by side-effects such as increased heart disease and cancer risk (4), and engagement with psychological therapies continues to be poor. Therefore, there is an unmet need to understand human sexual brain processing to help develop safer and more effective treatments.

The limbic system is an area of the brain known to control memory, emotions and behaviour, including those related to sexual desire and reproduction. Kisspeptin is a hormone which activates the reproductive axis leading to onset of puberty and stimulation of LH pulsatility. Kisspeptin is also widely expressed in the human limbic system and associated brain areas (5,6). When given to healthy men, kisspeptin reduces sexual aversion and negative mood with specific brain changes seen on functional magnetic resonance imaging (fMRI) (7). In animals, kisspeptin action at its receptor is required for olfactory-mediated sexual behaviour i.e. when male mice lose their kisspeptin receptors, they also lose the ability to preferentially sniff out a female partner (8). Mood is an important factor in psychosexual dysfunction and our recent study showing diminished negative mood in healthy men receiving kisspeptin (7) is consistent with animal data demonstrating antidepressant-like effects and modulation of fear response by kisspeptin via the serotonergic system (9,10).

Furthermore, kisspeptin also excites nerve fibres in specific memory formation areas within the hippocampus and amygdala of the limbic system (11). Cognitive testing in mice shows that administration of kisspeptin directly into the brain enhances learning, object recognition and memory consolidation (12,13). In addition, kisspeptin is reported to prevent Amyloid—β peptide neurotoxicity in vitro, which is the key mechanism for the development of Alzheimer's disease (14). Following on from these promising animal data, it is imperative to undertake human studies in order to assess whether kisspeptin has similar effects on human brain processing. Findings from these studies will deepen our understanding of human behaviour and cognitive processes with the aim of developing potential future therapies for psychosexual disorders and other conditions.

Objective

This study is designed to investigate the physiological effect of the naturally-occurring hormone kisspeptin on human brain processing compared to placebo (vehicle) using functional magnetic resonance imaging (fMRI).

Chief Investigator

The Chief Investigator is Professor Waljit S. Dhillo, Professor of Endocrinology and Metabolism, Department of Investigative Medicine, Imperial College London.

Kisspeptin

Kisspeptin is a naturally occurring hormone, which is known to play a critical physiological role in human fertility. Kisspeptin has been given safely to over 500 male and female participants in intravenous and subcutaneous form without side effects (15–18). The dose of kisspeptin administered in this study will not exceed 0.72nmol/kg/min. This dose has previously been demonstrated to safely produce a biological effect without untoward side effects (18). Similarly, the dose of intranasal kisspeptin will not exceed 0.72nmol/kg/min.

As per the MHRA algorithm, “Is it a clinical trial?”, the proposed study is not a clinical trial but rather an investigation of the physiological roles of kisspeptin and how it can modulate sexual and emotional brain processing in response to different stimuli. Vehicle administration will consist of gelofusine or normal saline. Kisspeptin and vehicle administration will not exceed 105 minutes duration.

Functional Brain MRI scan

Magnetic resonance imaging (MRI) is a safe and established imaging technique, which has been used routinely in hospitals throughout the world for over 30 years. MRI does not involve the administration of radiation, and has no associated health risks. Functional MRI (fMRI) is a type of MRI that is used to determine which areas of the brain have greatest blood flow as a surrogate marker of brain function. fMRI uses blood oxygen level dependent (BOLD) analysis, which requires no contrast agent or drug administration. The MRI scanner contains a very strong magnetic field and individuals with any type of metal implanted in their body which could move in this field cannot undergo magnetic resonance imaging for safety reasons. This will be carefully checked for and such individuals excluded at the time of initial screening. MRI safety criteria will also be confirmed by MRI staff prior to each scan. In some circumstances, participants may attend for a pre-study acclimatisation visit during which they will undergo an anatomical brain MRI.

Participants will have the opportunity to watch a video about what to expect in the scanner at their screening visit. Participants lie supine in the scanner with their head placed in a padded head coil for support. At all times the participant will have access to an alarm button and will be able to verbally communicate with the investigator at the scanning console in-between scans. Maximum scan time per study visit will be 75 minutes, starting after administration of kisspeptin or vehicle. Participants will be able to communicate with the researchers throughout the scan and will receive reassurance as required.

All scans will be formally reported by a consultant neuroradiologist. Very rarely, abnormalities may be detected that warrant further investigation. In this situation the report would be communicated to the participant’s GP, who will arrange the appropriate specialist referral. A significant abnormality would lead to exclusion from further participation in the study.

Participants

Study participants will be recruited from print and online advertisements (including local and regional newspapers). Suitable participants will be allocated to the following groups:-

Groups A and B: Healthy males (A) and females (B).

Group C and D: Males (C) and females (D) with mild to moderate low mood defined by Patient Health Questionnaire 9 (PHQ9) scores between 5-15 (19).

Group E and F: Males (E) and females (F) with low sexual desire defined by Diagnostic and Statistical Manual of Mental Disorders (DSM) V criteria.

Participant Consent and Safety
Potential participants who respond to the study advertisements will be sent a participant information sheet and invited to attend a screening visit at our Clinical Research Facility at Hammersmith Hospital or St Mary's Hospital.

During the screening visit, full informed consent will be obtained before any medical assessment takes place. Participants will be informed that they are free to withdraw from the study at any time.

After consent has been given, suitability to enter the study will be assessed with a full medical history. All participants, including those with no past history of psychological and psychosexual disorders will be screened for these conditions with a careful psychosexual history and use of standardised, validated psychometric questionnaires (including PHQ9, GAD7, IIEF, SCI-M, SDI, SCOFF and DEBQ). Any new diagnoses that are made will be discussed with the participant by one of the study doctors and if the participant consents, their GP will be contacted to arrange ongoing management or specialist referral as appropriate. If participants still wish to take part in the study in spite of a new diagnosis of low mood or low sexual desire, then they will be offered a place in the relevant study group provided they meet all inclusion and exclusion criteria. Severe features of any mental health disorder e.g. PHQ9 score > 15 indicating severe depression, will be discussed urgently as appropriate with their GP or Imperial College Healthcare NHS Psychiatry Liaison team.

The screening visit will also involve a physical examination (including an ECG) and blood tests for sex hormone levels, metabolic parameters and to assess for any systemic illness. A urine pregnancy test will be conducted to exclude pregnancy in women of childbearing age.

Inclusion Criteria for All Participants

- Aged 18 – 70 years
- Right handed
- Non-smoker
- Heterosexual orientation

Exclusion Criteria for All Participants

- History of any medical, psychological or other condition, or use of any medications, including over-the-counter products, which, in the opinion of the investigators, would either interfere with the study or potentially cause harm to the participant.
- Medical or psychological conditions that would impair their ability to participate reliably in the study or give informed consent.
- Pregnancy and/or breastfeeding.
- Any implanted material in the body that would preclude magnetic resonance imaging (MRI) for safety reasons.
- Inability to tolerate MRI scanning: such participants may be invited to take part in two non-fMRI study visits involving sensory, emotional and cognitive tasks (detailed below).
- Without access at home to a telephone, or other factor likely to interfere with ability to participate reliably in the study.
- History of hypersensitivity to any of the components administered.
- Treatment with an investigational drug within the preceding two months.
- Those who have or intend to donate blood or blood products within three months before or following study completion.
- A history of major haematological, renal, thyroid or hepatic abnormalities or significant cardiovascular disease.
- A history of cancer.

Primary Outcome:

Changes in task-related blood oxygen level dependent (BOLD) activity on fMRI when participants are administered kisspeptin compared to vehicle in each study group (A-F).

Secondary Outcomes:

- Changes in hormones such as luteinising hormone (LH), follicle-stimulating hormone (FSH), kisspeptin, oestradiol, testosterone, cortisol, and oxytocin during kisspeptin administration compared to vehicle.
- Changes in psychometric and behavioural parameters (including sexual, emotional and cognitive domains) during kisspeptin administration compared to vehicle.
- Correlation of changes in fMRI BOLD activity with psychometric and behavioural parameters during kisspeptin administration compared to vehicle.

Study Design: This project involves a randomised, two-way crossover placebo (vehicle) controlled study requiring 35 participants in each study group (A to F). Following their screening visit, suitable participants will attend for two fMRI study visits (one for kisspeptin and one for vehicle administration). Participants may also be invited to attend for two additional non-fMRI study visits (similarly one for kisspeptin and one for vehicle administration). Participants may consent for the two fMRI or two non-fMRI study visits or all four study visits. Each visit will last up to 4 hours to include preparation and post-study monitoring.

Pre-Study Visits: Participants will be asked to abstain from alcohol and caffeine from midnight before their study visits and to consume a normal breakfast on their study days. All study visits will start in the morning to ensure peak basal reproductive hormone levels. In addition, participants will be asked to abstain from sexual activity from midnight before their study visits, as sexual activity prior to the study can result in changes in testosterone levels and residual limbic brain activity (7).

fMRI Study Visits: Both study visits will take place at the Invicro Centre for Imaging Sciences and all participants (groups A to F) will receive the same protocol (see figure 1) during each study visit.

Figure 1 - Protocol Outline

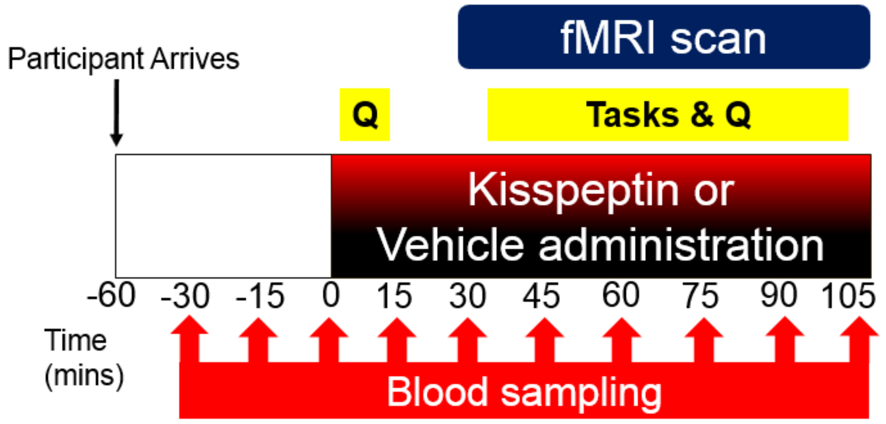

Kisspeptin will be administered during one study visit and vehicle will be administered during the other study visit. Participants will be blinded to the identity of kisspeptin or vehicle and the order of study visits will be randomised by an independent investigator (using Research Randomizer, www.randomizer.org).

On arrival, participants will change into loose hospital scrubs and lie supine for 30 minutes to relax. Female participants will have their urine tested at the beginning of each study visit to ensure that they are not pregnant. The participant will complete an MRI safety questionnaire on each visit before entering the MRI suite.

Blood samples will be taken at regular intervals through an intravenous cannula (not more frequently than 15 minute intervals) for measurement of LH, FSH, kisspeptin, testosterone, oestradiol, cortisol and oxytocin to ensure baseline reproductive hormone levels are equivalent between study visits and to observe any changes during each study. At least two clinically trained investigators will be present throughout each study visit. Participants will have their blood pressure recorded on arrival and on completion of the visit, with their pulse measured at regular intervals throughout MRI scanning.

At t = 0 minutes, kisspeptin or vehicle will be administered (maximum administration time 105 minutes). At t = 30 minutes, the fMRI scan will begin (maximum scan time 75 minutes). During fMRI scanning participants will be presented with standard, validated tasks designed to activate sensory, emotional, cognitive and reproductive brain structures so kisspeptin's modulation of this activity can be assessed.

i) *Sensory Tasks*

These may include olfactory tasks in which participants will be presented with validated odours in a randomised order. During the task, participants may be asked to state whether they can smell anything, and to rate the intensity and the pleasantness of the odour on visual analog scales which will be projected on a computer screen in the MRI scanner (20,21).

ii) *Emotional Tasks*

These may include facial perception tasks (22,23) and viewing of emotive images (7) to determine if changes in brain activity modulated by kisspeptin affect resultant behaviour.

iii) *Cognitive Tasks*

These may involve general mental capacity tasks, evaluating a range of basic sensory and cognitive modalities. A battery fMRI task (7) may be employed to assess visual, motor, auditory language and calculation functions; and N-back tasks may be used to assess working memory (24).

iv) *Penile Tumescence Tasks (male participants)*

To provide functional significance for the fMRI effects observed during the relevant tasks, simultaneous penile tumescence using an MRI-compatible pneumatic device may be measured. This will be undertaken in accordance with validated and published methods for assessing penile tumescence in an fMRI environment (25,26).

At the beginning and the end of each study, participants will complete standardised, validated psychometric questionnaires (including BIS/BAS, IIEF, SDI-2, Self-Evaluation Questionnaire STAI Form Y-2, SQOL-M, Subjective Happiness Scale, Satisfaction with Life Scale, SADI, POMS2A, PANAS, PLS, Love Attitudes Scale, Three-Factor Eating Questionnaire, FCQ-S15 and Visual Analog Scale). These will be correlated with the fMRI data to provide further functional relevance for the effects observed in the sensory, emotional and cognitive tasks above.

Non-fMRI Study Visits: Given it is recognised that the MRI scanner environment impacts on perceptual decision-making with increased error rates and less attentional focus during fMRI tasks

compared with the same tasks performed outside of the scanner (27), participants may be invited to take part in two non-fMRI study visits.

Both study visits will take place at the Clinical Research Facility at Charing Cross Hospital. With the exception of not undergoing an fMRI scan, all participants (groups A to F) will receive the same protocol as detailed in the section titled ‘fMRI Study Visits’ at each study visit.

Blood samples will be taken at regular intervals through an intravenous cannula (not more frequently than 15 minute intervals) for measurement of LH, FSH, kisspeptin, testosterone, oestradiol, cortisol and oxytocin to ensure baseline reproductive hormone levels are equivalent between study visits and to observe any changes during each study. At least two clinically trained investigators will be present throughout each study visit. Participants will have their blood pressure and heart rate recorded on arrival and at regular intervals throughout the study visit.

At t = 0 minutes, kisspeptin or vehicle will be administered (maximum administration time 105 minutes). At t = 30 minutes, the participants will start the tasks (maximum allocated time 75 minutes). During the study visits participants will be presented with standard, validated sensory, emotional and cognitive tasks so kisspeptin’s modulation of human brain processing can be assessed.

i) *Sensory Tasks*
These may include olfactory tasks in which participants will be presented with validated odours in a randomised order. During the task, participants may be asked to state whether they can smell anything, and to rate the intensity and the pleasantness of the odour on visual analog scales.

ii) *Emotional Tasks*
These may include facial perception tasks (22,23) and viewing of emotive images (7) and videos to determine if kisspeptin affects resultant behaviour as assessed using participant rating scores on a visual analogue scale.

iii) *Cognitive Tasks*
These may involve general mental capacity tasks, evaluating a range of basic sensory and cognitive modalities to assess visual, motor, auditory language and calculation functions; and N-back tasks may be used to assess working memory (24).

Blood Volumes
At the screening visit, 10ml of blood will be taken for screening blood tests detailed above. During each study visit, 10 blood samples will be taken at 15 minute intervals (see Figure 1). A maximum of 14 ml will be taken for each sample (3ml for LH and FSH, 3ml for testosterone and oestradiol, 3ml for kisspeptin, 3ml for oxytocin, 2ml for cortisol). Study visits will be scheduled at least 1 week apart and total blood volume taken during the study will not exceed 290mls (includes screening and both study visits). For participants completing four study visits, an additional 280mls of blood will be taken (total 570mls) with a minimum time of six weeks between the fMRI and non-fMRI study visits. For comparison, the volume of blood taken during a single standard blood donation session is 470mls (24).

Safety of Participants during the Study

Participants will remain in the investigation unit for a short period of observation after termination of the study visit. During the study, at least one experienced physician will monitor the participant at all times with clinical observations (pulse regularly and blood pressure before and after fMRI scanning, and regular heart rate and blood pressure monitoring throughout the non-fMRI study visits). Participants will be encouraged to report any unusual or unpleasant sensation to the investigators immediately. Any significant adverse effects will lead to withdrawal of the individual and any serious adverse effects will terminate the whole study.

Throughout the study there will be at least one physician available 24 hours per day via a direct line, with a second physician on back up, and a secondary direct line to one of the senior physicians. Although we do not anticipate any serious adverse effects, participants will be provided with contact numbers and clear instructions that, if they feel unwell, they should call us. Kisspeptin is rapidly cleared (18) and participants would not be anticipated to experience any delayed effects on completion of the study visit.

Informed, written consent will be obtained before initiation of the study and it will be made clear to participants that they are free to withdraw from the study at any time without providing any reason. Participants will be given an adverse event form to fill in if they have untoward symptoms (using Imperial College London standard template). Any possible adverse event will then be reviewed with the senior clinicians (principally Professor Dhillo). Any significant adverse effects would lead to withdrawal of the individual and any serious adverse effects would terminate the whole trial. Any serious adverse event occurring during the study will be reported to the sponsor (Imperial College London). Serious adverse events that are unexpected or suspected to be related to study procedures will also be reported to the ethics committee as well as to the data monitoring committee. The data monitoring committee will be convened prior to initiation, and an internal audit will be completed at the end of the study.

If a participant loses capacity during the study then they would be withdrawn from further participation in the study. However, any data or samples that we had already collected when they were able to give consent would be used in the study. We will seek their informed written consent for this at the screening visit.

Statistics and Data Analysis

Mr Paul Bassett (independent statistical consultant) calculated the sample size required. Our previous work (7) demonstrates kisspeptin enhances blood oxygen level dependent (BOLD) signal change in the amygdala by mean 0.74% and standard deviation 0.38% compared to vehicle (mean 0.48%, standard deviation 0.51%) and we anticipate a similar response in this study. Using these data, with alpha 0.05, power 0.8, and assuming correlation between means of 0.40, a power calculation has been performed resulting in n of 31. This is in line with previous fMRI studies and empirically-derived estimates of optimal sample sizes in fMRI studies (26). To allow for natural variation in responses, drop-out and exclusion rate (e.g. *a priori* head movement > 3mm) of 10%, we plan to recruit 35 participants per study group (A – F) giving 210 participants in total.

Handling and storage of Samples and Data

The samples taken during the screening visit are labelled with the subject's name and hospital number, in a manner similar to other NHS samples. Samples taken during the study visits will be coded. Only the researchers involved in the study will have access to the codes. This is necessary to identify samples in order to correlate hormone levels found within the samples to clinical details of the donors. The samples will be transferred to the Department of Investigative Medicine at Imperial College for analysis. Blood samples will be stored in the Department of Investigative Medicine, Commonwealth Building, Hammersmith Hospital, Du Cane Road, London. Only members of the research team will have access to the samples and all results will be stored and analysed on password-protected university computers. fMRI raw data will be stored on secure, password-protected computers at Invicro Centre for Imaging Sciences prior to analysis by study researchers. Data will only be accessed by the researchers involved in the study. With the participant's consent, samples may be stored at Imperial College London for future ethically approved studies.

Regulatory issues

Ethical approval: The Chief Investigator will obtain approval from London Riverside Research Ethics Committee. The study has been submitted for HRA approval. The study will be conducted in

accordance with the recommendations for physicians involved in research on human subjects adopted by the 18th World Medical Assembly, Helsinki 1964 and later revisions.

Consent: Written consent to enter the study will be sought from each participant only after a full explanation has been given, an information leaflet offered and time allowed for consideration. The right of the participant to refuse to participate without giving reasons must be respected. After the participant has entered the study the clinician remains free to give alternative treatment to that specified in the protocol at any stage if he/she feels it is in the participant's best interest, but the reasons for doing so should be recorded. In these cases the participants remain within the study for the purposes of follow-up and data analysis. All participants are free to withdraw at any time without giving reasons and without prejudicing further treatment.

Confidentiality: Full confidentiality will be maintained for all participants. Their data will only be accessed by members of the research team. All samples will be coded, and these codes will only be accessible to researchers directly involved with the study. All records will be maintained in secure containment for 10 years after the study.

Indemnity: Imperial College London holds negligent harm and non-negligent harm insurance policies which apply to this study.

Sponsor: Imperial College London will act as the main Sponsor for this study. Delegated responsibilities will be assigned to the NHS trusts taking part in this study.

Funding: This study will be funded from Professor Waljit Dhillo's NIHR Research Professorship. Participants will receive £100 per completed study visit to cover expenses including travel costs, time off work and lost earnings. The investigators do not receive any payment for this study.

Audits: The study may be subject to inspection and audit by Imperial College London under their remit as sponsor and other regulatory bodies to ensure adherence to GCP and the NHS Research Governance Framework for Health and Social Care (2nd edition).

Study Management

The day-to-day management of the study will be co-ordinated through Dr Lisa Yang, Dr Edouard Mills, Miss Lysia Demetriou, Dr Matt Wall and Dr Alexander Comninos.

Publication Policy

We aim to disseminate data generated during the study via publication in peer reviewed medical journals, presentation at conferences and publication in the lay press as appropriate. During publication the data will be completely anonymised and no personal information will be published. Participant confidentiality will be maintained throughout. Participants and their GPs (with the participant's consent) will be provided with a copy of published data should they wish

References

1. Laumann EO, Paik A, Rosen RC. Sexual dysfunction in the United States: prevalence and predictors. Jama. 1999;281(6):537–44.

2. Khademi A, Alleyassin A, Amini M, Ghaemi M. Evaluation of sexual dysfunction prevalence in infertile couples. J Sex Med. 2008;5(6):1402–10.

3. Wilson ECF, McKeen ES, Scuffham PA, Brown MCJ, Wylie K, Hackett G. The cost to the United Kingdom National Health Service of managing erectile dysfunction: The impact of sildenafil and prescribing restrictions. Pharmacoeconomics. 2002;20(13):879–89.

4. Basaria S, Coviello AD, Travison TG, Storer TW, Farwell WR, Jette AM, et al. Adverse events associated with testosterone administration. N Engl J Med. 2010;363(2):109–22.

5. Muir AI, Chamberlain L, Elshourbagy NA, Michalovich D, Moore DJ, Calamari A, et al. AXOR12, a novel human G protein-coupled receptor, activated by the peptide KiSS-1. J Biol Chem. 2001;276(31):28969–75.

6. Pineda R, Plaisier F, Millar RP, Ludwig M. Amygdala Kisspeptin Neurons: Putative Mediators of Olfactory Control of the Gonadotropic Axis. Neuroendocrinology. 2016;104(3):223-238.

7. Comninos AN, Wall MB, Demetriou L, Shah AJ, Clarke SA, Narayanaswamy S, et al. Kisspeptin modulates sexual and emotional brain processing in humans. J Clin Invest. 2017;127(2):709-719.

8. Kauffman AS, Park JH, McPhie-Lalmansingh AA, Gottsch ML, Bodo C, Hohmann JG, et al. The Kisspeptin Receptor GPR54 Is Required for Sexual Differentiation of the Brain and Behavior. J Neurosci. 2007;27(33):8826-35.

9. Tanaka M, Csabafi K, Telegdy G. Neurotransmissions of antidepressant-like effects of kisspeptin-13. Regul Pept. 2013;180:1–4.

10. Nathan FM, Ogawa S, Parhar IS. Kisspeptin1 modulates odorant-evoked fear response via two serotonin receptor subtypes (5-HT1A and 5-HT2) in zebrafish. J Neurochem. 2015;133(6):870–8.

11. Arai AC. The role of kisspeptin and GPR54 in the hippocampus. Peptides. 2009;30(1):16–25.

12. Telegdy G, Adamik A. The action of kisspeptin-13 on passive avoidance learning in mice. Involvement of transmitters. Behav Brain Res. 2013;243:300–5.

13. Jiang JH, He Z, Peng YL, Jin WD, Wang Z, Han RW, et al. Kisspeptin-13 enhances memory and mitigates memory impairment induced by Abeta1-42 in mice novel object and object location recognition tasks. Neurobiol Learn Mem. 2015;123:187–95.

14. Milton NG, Chilumuri A, Rocha-Ferreira E, Nercessian AN, Ashioti M. Kisspeptin prevention of amyloid-beta peptide neurotoxicity in vitro. ACS Chem Neurosci. 2012;3(9):706–19.

15. Dhillo WS, Chaudhri OB, Patterson M, Thompson EL, Murphy KG, Badman MK, et al. Kisspeptin-54 Stimulates the Hypothalamic-Pituitary Gonadal Axis in Human Males. J Clin Endocrinol Metab. 2005;90(12):6609–15.

16. Dhillo WS, Chaudhri OB, Thompson EL, Murphy KG, Patterson M, Ramachandran R, et al. Kisspeptin-54 Stimulates Gonadotropin Release Most Potently during the Preovulatory Phase of the Menstrual Cycle in Women. J Clin Endocrinol Metab. 2007;92(10):3958–66.

17. Jayasena CN, Abbara A, Comninos AN, Nijher GM, Christopoulos G, Narayanaswamy S, et al. Kisspeptin-54 triggers egg maturation in women undergoing in vitro fertilization. J Clin Invest. 2014;124(8):3667–77.

18. Jayasena CN, Nijher GMK, Comninos AN, Abbara A, Januszewki A, Vaal ML, et al. The Effects of Kisspeptin-10 on Reproductive Hormone Release Show Sexual Dimorphism in Humans. J Clin Endocrinol Metab. 2011;96(12):E1963–72.

19. Kroenke K, Spitzer RL, Williams JB. The PHQ-9: validity of a brief depression severity measure. J Gen Intern Med. 2001;16(9):606–13.

20. Huh J, Park K, Hwang IS, Jung SI, Kim HJ, Chung TW, et al. Brain activation areas of sexual arousal with olfactory stimulation in men: a preliminary study using functional MRI. J Sex Med. 2008;5(3):619–25.

21. Zhou W, Yang X, Chen K, Cai P, He S, Jiang Y. Chemosensory communication of gender through two human steroids in a sexually dimorphic manner. Curr Biol. 2014;24(10):1091–5.

22. Perrett DI, Lee KJ, Penton-Voak I, Rowland D, Yoshikawa S, Burt DM, et al. Effects of sexual dimorphism on facial attractiveness. Nature. 1998;394(6696):884-7.

23. Striepens N, Matusch A, Kendrick KM, Mihov Y, Elmenhorst D, Becker B, et al. Oxytocin enhances attractiveness of unfamiliar female faces independent of the dopamine reward system. Psychoneuroendocrinology. 2014;39(1):74–87.

24. Ragland JD, Turetsky BI, Gur RC, Gunning-Dixon F, Turner T, Schroeder L, et al. Working memory for complex figures: an fMRI comparison of letter and fractal n-back tasks. Neuropsychology. 2002;16(3):370–9.

25. Arnow BA, Desmond JE, Banner LL, Glover GH, Solomon A, Polan ML, et al. Brain activation and sexual arousal in healthy, heterosexual males. Brain. 2002;125(5):1014-23.

26. Cera N, di Pierro ED, Sepede G, Gambi F, Perrucci MG, Merla A, et al. The Role of Left Superior Parietal Lobe in Male Sexual Behavior: Dynamics of Distinct Components Revealed by fMRI. J Sex Med. 2012;9(6):1602-12.

27. van Maanen L, Forstmann BU, Keuken MC, Wagenmakers EJ, Heathcote A. The impact of MRI scanner environment on perceptual decision-making. Behav Res Methods. 2016;48(1):184–200.

28. NHS Blood and Transplant. After your donation. Vol. 2016.

29. Murphy K, Garavan H. An empirical investigation into the number of subjects required for an event-related fMRI study. Neuroimage. 2004;22(2):879–85.
