# Supplementary material for: Effects of Kisspeptin on Sexual Brain Processing and Penile Tumescence in Men With Hypoactive Sexual Desire Disorder: A Randomized Clinical Trial
Source: JAMA Netw Open. 2023 Feb 3;6(2):e2254313. doi: 10.1001/jamanetworkopen.2022.54313 (PMC9898824; doi:10.1001/jamanetworkopen.2022.54313)
Supplement: Supplement 2. — eMethods. eReferences eFigure 1. Participant Recruitment and Flow Summary eFigure 2. Effects of Kisspeptin Administration on Hormone Levels eFigure 3. Effects of Kisspeptin Administration on Sexual Arousal and Desire Inventory Scores eFigure 4. Effects of Kisspeptin Administration on Positive and Negative Mood, Anxiety, and Nonsexual Attention eFigure 5. Effects of Kisspeptin Administration on Blood Pressure and Heart Rate Measurements eFigure 6. Group Mean Analysis for Main Effects of the Short Videos Task in All Participants and Both Treatments (Kisspeptin and Placebo) Demonstrating Effective Task Design eFigure 7. Group Mean Analysis for Brain Areas Associated With Increasing Penile Tumescence During the Long Video Task in All Participants and Both Treatments (Kisspeptin and Placebo) Demonstrating Effective Task Design eFigure 8. Group Mean Analysis of Brain Areas Associated With Increasing Subjective Arousal During the Long Video Task in All Participants and Both Treatments (Kisspeptin and Placebo) Demonstrating Effective Task Design eTable 1. Participant Baseline Clinical and Psychometric Characteristics at the Beginning of the Kisspeptin and Placebo Visits eTable 2. Clusters With Enhanced Activation or Deactivation by Kisspeptin on Whole-Brain Analysis eTable 3. Effect Sizes for Brain Regions With Significantly Enhanced Activation or Deactivation by Kisspeptin Administration eTable 4. Safety Outcomes [file jamanetwopen-e2254313-s002.pdf]

Supplementary Online Content

Mills EG, Ertl N, Wall MB, et al. Effects of kisspeptin on sexual brain processing and penile tumescence in men with hypoactive sexual desire disorder: a randomized clinical trial. *JAMA Netw Open*. 2023;6(2):e2254313.
doi:10.1001/jamanetworkopen.2022.54313

eMethods.

eReferences

eFigure 1. Participant Recruitment and Flow Summary

eFigure 2. Effects of Kisspeptin Administration on Hormone Levels

eFigure 3. Effects of Kisspeptin Administration on Sexual Arousal and Desire Inventory Scores

eFigure 4. Effects of Kisspeptin Administration on Positive and Negative Mood, Anxiety, and Nonsexual Attention

eFigure 5. Effects of Kisspeptin Administration on Blood Pressure and Heart Rate Measurements

eFigure 6. Group Mean Analysis for Main Effects of the Short Videos Task in All Participants and Both Treatments (Kisspeptin and Placebo) Demonstrating Effective Task Design

eFigure 7. Group Mean Analysis for Brain Areas Associated With Increasing Penile Tumescence During the Long Video Task in All Participants and Both Treatments (Kisspeptin and Placebo) Demonstrating Effective Task Design

eFigure 8. Group Mean Analysis of Brain Areas Associated With Increasing Subjective Arousal During the Long Video Task in All Participants and Both Treatments (Kisspeptin and Placebo) Demonstrating Effective Task Design

eTable 1. Participant Baseline Clinical and Psychometric Characteristics at the Beginning of the Kisspeptin and Placebo Visits

eTable 2. Clusters With Enhanced Activation or Deactivation by Kisspeptin on Whole-Brain Analysis

eTable 3. Effect Sizes for Brain Regions With Significantly Enhanced Activation or Deactivation by Kisspeptin Administration

eTable 4. Safety Outcomes

This supplementary material has been provided by the authors to give readers additional information about their work.

© 2023 Mills EG et al. *JAMA Network Open.*

eMethods

Participant screening

Right-handed and heterosexual men concerned and/or distressed by low sexual desire were invited to take part via advertisements placed online, in local newspapers and across the Transport for London infrastructure network. Right-handed (i.e., the dominant hand in >90% of the human population) rather than left-handed participants were included, which is the convention in fMRI studies as brain lateralisation of neural functioning significantly differs between left- and right-handed people[1]. Participants were screened initially using a self-reported questionnaire and telephone screening appointment. Those potentially meeting eligibility were invited to attend a detailed face-to-face medical and clinical examination screening visit during which they were interviewed by an experienced physician with expertise in sexual medicine (EM/DG) to ascertain a diagnosis of acquired and generalisable HSDD (in accordance with the DSM-V diagnostic criteria[2]). Participants completed a battery of psychometric questionnaires, including the 'Sexual Concerns Inventory-Male' (SCI-M) and 'Sexual Desire Inventory' (SDI) to assess sex-related distress, with the scores obtained **(Table 1)** equivalent to those previously published in men with HSDD[3]. Normal erectile function was confirmed using the 'International Index of Erectile Function'[4]. Active depression and anxiety were ruled out using the 'Patient Health Questionnaire-9' (PHQ-9) and 'Generalised Anxiety Disorder Assessment-7' (GAD-7) instruments, respectively **(Table 1)**. Exclusive heterosexuality was determined by a Kinsey score of 0.

Additional inclusion criteria included age 18-70 years, BMI 18-30 kg/m^2, being in a stable, monogamous and communicative relationship for ≥ 6months prior to their screening appointment, free of current or past physical or psychiatric illness, free of current medications or psychoactive substances (prescribed or illicit) for ≥ 6months, experience of viewing sexually explicit material, and normal or corrected-to-normal vision. Men were excluded if they had a history of unresolved sexual trauma, abuse or aggression, use of medications (prescribed or over the counter) or herbal preparations to enhance sexual desire, arousal or performance, or had contraindication to MRI scanning. The following blood tests were assessed at screening to confirm health status and exclude an underlying endocrinopathy: full blood count, renal function, liver function, bone profile, thyroid hormone profile, LH, FSH, testosterone, and sex hormone binding globulin. All participants had normal basal reproductive hormone levels **(Table 1)** and had structurally normal brains on MRI as reported by a neuroradiologist (independent of the fMRI data analysts).

Based on our sample size calculation (see **Sample Size**), 37 participants were recruited. Two participants were excluded due to excessive head motion during the scan (*a priori* criteria; >2mm translation), two participants were excluded due to abnormal structural findings on the MRI and one participant did not complete the fMRI tasks, resulting in a final group of 32 men with HSDD **(Supplemental eFigure 1)**.

Randomisation and blinding

The randomisation sequence was generated using an independent web-based randomisation service (www.randomizer.org) by author EM prior to recruitment of the first participant. This randomly allocated participant numbers in a 1:1 ratio to sequence Placebo-Kisspeptin or Kisspeptin-Placebo. After recruitment, participants were allocated an individualised participant number in sequential order. The assignment schedule was held centrally at Imperial College London and concealed from the study team at the Invicro London Clinical Imaging Centre. All study participants, research nurses, radiographers and fMRI data analysts were blinded to the treatment sequence. Intravenous infusions of kisspeptin or placebo were prepared by author LT, were identical in appearance, volume and rate, and infused in identical syringe pumps.

Study design

This was a randomised, double-blind, two-way crossover, placebo-controlled study **(Figure 1A)** with participants attending two study visits each (kisspeptin and placebo) at least seven days apart (to allow full wash out given that the half-life of kisspeptin-54 is 27.6 minutes in humans[5]). Participant recruitment took place at Imperial College Healthcare NHS Trust (St Mary's and Charing Cross Hospitals, London, UK), with all study visits performed at the Invicro London Clinical Imaging Centre (Hammersmith Hospital Campus, London, UK) from January 2021 to September 2021. The cross-over design, in which participants acted as their own control, minimised interparticipant variation and enhanced power. Additionally, the study was counterbalanced, which along with the washout period was designed to minimise any possible crossover effects between study visits. All study visits commenced in the

© 2023 Mills EG et al. *JAMA Network Open.*

morning to control for circadian hormonal changes. Participants were asked to abstain from alcohol from midnight the night preceding each study visit, given that recent alcohol is known to affect fMRI task performance and brain structures implicated in motivation and behaviour control[6], as well as caffeine for the same period, as the vasoconstrictive effects of caffeine are known to alter fMRI BOLD activity[7]. Participants were also asked to abstain from all sexual activity from midnight preceding each study visit, given that recent sexual activity can affect testosterone levels[8] and is associated with a refractory period during which sexual stimuli fails to trigger sexual arousal[9]. Participants were asked to consume a normal breakfast before attending each visit, owing to the established overlap between hunger and food anticipation brain areas with those associated with sexual behaviour[10].

Kisspeptin-54 peptide and placebo

Kisspeptin-54 was synthesised by Bachem and purified by reverse-phase high-performance liquid chromatography. Vials of freeze-dried kisspeptin-54 were stored at -20°C and made up in Gelofusine (B. Braun, Germany) and infused (1 nmol/kg/hour) as previously described[11]. The kisspeptin dose was selected to ensure steady-state levels of circulating kisspeptin from 30 to 75 minutes (during the fMRI data collection period) while avoiding downstream testosterone increases which are known to occur after 90 minutes following kisspeptin exposure in humans[11–16]. Based on our previous experience, we also know this dose is effective in healthy men to enhance sexual brain processing[11,13,14]. Placebo (Gelofusine) was administered at a rate equivalent to the kisspeptin infusion. Both infusions were administered via a Medrad Spectris Solaris MRI-compatible injection system (Bayer AG, Germany) controlled from a remote panel in the control room.

Hormonal assays

Blood samples were collected to measure circulating plasma kisspeptin immunoreactivity, and serum reproductive hormone and cortisol levels at the time points depicted in **Figure 1A**. Plasma kisspeptin immunoreactivity was measured using an established radioimmunoassay[11]. The intra- and interassay coefficients of variation were 8.3 and 10.2%, respectively, and the limit of detectability was 2 pmol/L[11]. Serum LH, FSH and testosterone were measured using automated chemiluminescent immunoassays (Abbott Diagnostics, UK). Serum cortisol was measured using automated delayed one-step immunoassay (Abbott Diagnostics, UK). Intra-assay and inter-assay coefficients of variation were as follows: LH, < 5%; FSH, < 5%; total testosterone, < 5%; cortisol, < 10%. Limits of detection for each assay were as follows: LH in international units per litre (IU/L), 0.07; FSH in IU/L, 0.05; total testosterone in nanomoles per litre (nmol/L), 0.05; cortisol in nmol/L, 22.

Behavioural assessments

At their first study visit, participants completed a battery of well-established validated psychometric questionnaires to assess relevant baseline traits prior to administering kisspeptin or placebo **(Table 1)**. The State-Trait Anxiety Inventory (STAI-Y2-Trait) was used to exclude anxiety traits in our cohort, with all scores within normal range. The Behavioural Inhibition and Activation System Scales were used to assess sensitivity to anticipation of punishment and reward. The Subjective Happiness Scale was used to assess overall subjective happiness. The Sexual Quality of Life-Men was used to assess the impact of sexual dysfunction on quality of life. The Satisfaction with Life Scale was used to assess satisfaction with life more generally.

A second battery of validated psychometric questionnaires was used to assess behavioural parameters in the current moment. The State-Trait Anxiety Inventory (STAI-Y1-State) was used to assess the effect of kisspeptin and placebo on anxiety in the current moment, with no difference observed (**Supplemental eFigure 4C**). The multidimensional, descriptor-based Sexual Arousal and Desire Inventory (SADI) was used to assess sexual arousal and desire[17], as previously employed in other interventional neuroimaging studies[11]. The SADI questionnaire comprises of multiple descriptors to evaluate physiological (e.g., flushed, tingling in genital area), evaluative (e.g., hard, happy), negative/aversive (e.g., aversion, repulsion), and motivational (e.g., horny, naughty) domains of the subjective experience of sexual desire and arousal in the current moment[17], with no difference observed at a domain level **(Supplemental eFigure 3A-D).** The Positive and Negative Affect Schedule (PANAS) contains 20-items assessing positive and negative emotions and feelings, with no difference observed **(Supplemental eFigure 4A-B)**. Participants completed these questionnaires before and towards the end of kisspeptin or placebo administration on their first and second study visits **(Figure 1A)**. Participants also completed the d2 Test of Attention during their infusions to exclude

© 2023 Mills EG et al. *JAMA Network Open.*

differences in non-sexual attention between kisspeptin or placebo administration, with no difference observed **(Supplemental eFigure 4D)**.

Sexual stimuli selection

To select the sexual videos for the short and long video fMRI tasks, 20 healthy heterosexual men participated in an independent focus group, in which they were asked to rate 60 short videos (lasting 20-seconds each) and six long videos (lasting eight minutes each) taken from commercial adult films. All the videos depicted heterosexual sexual activity between one man and one woman, with the short videos depicting exclusively sexual intercourse, and the long videos including a range of sexual activity. Each volunteer privately viewed and rated the video segments according to sexual arousability. The 10 short videos and one long video with the highest scores were selected and formed the stimulus sets for the fMRI tasks. This procedure was comparable to that described in a previous neuroimaging study examining brain processing in patients with HSDD, in which a focus group of healthy individuals with normal sexual function selected the sexual videos which had the greatest chance of inducing arousal[18]. To ensure that participants were presented with sexual stimuli of equivalent sexual arousability, men were shown the same videos on both the kisspeptin and placebo visits. However, to minimise habituation, the short videos were presented in a different order on each visit. This approach is consistent with previous interventional fMRI studies examining hormone administration versus placebo on sexual brain activity in patients with HSDD[19,20].

MRI acquisition

Imaging data were acquired using a 3T Siemens Trio scanner with a 32-channel, phased-array head coil. Anatomical images were acquired at the start of each scan using a T1-weighted magnetisation prepared rapid gradient echo (MPRAGE) pulse sequence (1 mm isotropic voxels, repetition time [TR] = 2300 ms, echo time [TE] = 2.98 ms, flip angle = 9°, 160 slices, 256x256 in-plane FOV, bandwidth = 240Hz/pixel, GRAPPA acceleration = 2). For the acquisition of functional images (in both the video tasks), a multiband sequence with acceleration factor 2 (similar to the sequences previously validated in[21]) was used with the following parameters: 3 mm isotropic voxels, TR = 1250 ms, TE = 30 ms, flip angle = 80°, 44 axial slices, bandwidth = 2232Hz/pixel, GRAPPA acceleration = 2, 192x192 mm FOV. A pulse oximeter was used to monitor physiological data by means of a standard data-recording system (PowerLab, AD Instruments, New Zealand) in the control room.

fMRI procedure

During the MRI session, a series of anatomical and functional brain scans were performed. During the functional tasks, a mirror mounted on the head coil was used to view a screen at the rear of the scanner bore, onto which the stimuli were projected. To respond to the tasks, the participants were equipped with an MRI-compatible custom-made, button response box (short video task) or a scroll wheel (long video task) as detailed below.

Short video task: This task examined the early neural correlates of sexual arousal (i.e., cognitive, emotional and motivational components). This was a standard block-design task lasting 12 minutes. Participants were shown 20-second segments of sexually explicit videos (depicting one woman and one man engaging in sexual intercourse), alternating with neutral non-sexual videos (depicting a woman and man exercising) as a control. During the task, 10 different sexually explicit and 10 control videos were shown. To maintain alertness and task engagement, after each video participants were asked to rate their subjective level of arousal on a scale of 1 to 20 (1 = very sexually unarousing, 20 = very sexually arousing) using their index and middle fingers to move up and down a scale which they could see on the screen. The rating period lasted for five seconds and was followed by a 10-second blank grey screen which provided a baseline/rest condition. Participants rated the sexual videos as more arousing than the control exercise videos as expected, with no significant differences observed between kisspeptin and placebo visits.

Long video task: This task was used to study the brain areas activated during progression from general sexual arousal to a sustained sexual response and lasted eight minutes (plus a 10-second buffer period at the end (blank grey screen) to ensure capture of the latter portion of the brain response). Participants were shown a continuous eight minute long sexually explicit video depicting one woman and one man engaging in sexual activities (kissing, manual stimulation, oral sex, and vaginal intercourse). To ensure alertness and task engagement, participants were asked to rate their subjective level of arousal (on a continuous scale from “Not at all sexually aroused” to “Very sexually aroused”) in real time using an MR-compatible scroll wheel. Given that penile erection is the most robust marker of sexual arousal in men[22], we simultaneously recorded penile tumescence and the associated brain responses as detailed below.

© 2023 Mills EG et al. *JAMA Network Open.*

fMRI data analysis

All imaging analysis was performed using the Oxford Centre for Functional MRI of the Brain (FMRIB) Software Library (FSL) version 6.0 (www.fmrib.ox.ac.uk/fsl), using the FEAT (fMRI Expert Analysis Tool) as previously described[11,14,15]. Functional data were registered to a high-resolution structural image using FMRIB Linear Imaging Registration Tool (FLIRT). Then each participant's high-resolution image was registered to standard Montreal Neurological Institute (MNI) 152 space using FMIRIB Nonlinear Registration Tool (FNIRT). Pre-processing methods used the Brain Extraction Tool (BET), spatial smoothing (6 mm), temporal high-pass filtering (90 seconds for the short videos task, 100 seconds for the long video task) and motion correction. White Matter (WM) and Cerebro-Spinal Fluid (CSF) masks were created from each participant's brain-extracted anatomical scan using the FMRIB Automated Segmentation Tool (FAST).

In the first level analyses of the short video task, a general linear model (GLM), modelled the occurrence of the task stimuli with the onset timings of the sexual/control videos convolved with a gamma function to simulate the haemodynamic response function (HRF). The contrasts were defined by each stimulus condition compared to baseline and also comparing the two stimulus conditions of interest (sexual compared to control), which was the main outcome. As well as an extended set of head-motion parameters (six standard motion parameters, their temporal derivatives, plus squares of the previous[12]), further physiological denoising was performed by including the mean time series from the WM and CSF masks from each participant as regressors of no interest.

The long video task was modelled in two ways. Firstly, using a subjective measure of arousal which was the (down-sampled) time series derived from the participant moving the dial throughout the video. This was convolved with a gamma function and used as the main regressor of interest. Secondly using the objective measure of arousal which was the (down-sampled) time series derived from the penile tumescence data**.** The processed penile tumescence time-series was convolved with a gamma function and negatively phase shifted 20 seconds (i.e. shifted 20 seconds earlier in time) to account for the physiological time-lag between brain activity and the downstream vascular effects in the penis[23]. This phase-shifted time-series was then used as the main regressor of interest in the GLM. The same denoising set of regressors (extended motion parameters, plus mean WM and CSF signals) were included in both analyses as regressors of no interest. Both models also used simple positive contrasts on the main regressor of interest.

Group level models were analysed in FEAT using FMRIB's Local Analysis of Mixed Effects (FLAME-1). To test for differences between kisspeptin and placebo, a within-subjects paired one-sided *t*-test GLM was constructed. To provide validation of the procedures and examine task main effects an additional group average model was constructed which averaged across both drug conditions. Statistical maps were thresholded at $Z = 2.3$ and $P < .05$ (cluster-corrected for multiple comparisons). Cluster-based thresholding is a commonly applied method to correct for multiple tests in the standard mass-univariate approach[24,25] and provides good sensitivity and adequate control of false positives[26]. Group-mean analyses (including both kisspeptin and placebo visits) for both tasks resulted in similar patterns of brain activity to previous studies which have examined sexual arousal[22,23,27–32], demonstrating that the tasks worked effectively **(Supplemental eFigure 6-8).**

To assess behavioural and functional relevance for our brain data, correlation analyses between behavioural parameters (from psychometric questionnaires) and neuroimaging data (based on *a priori*–selected brain regions) were performed. A set of *a priori*–selected brain regions defined in standard stereotactic space using the Harvard-Oxford atlases (https://fsl.fmrib.ox.ac.uk/fsl/fslwiki/) was used to extract data for ROI analyses. Regions were selected to include limbic brain areas that express kisspeptin receptors in humans[33–35] and involved in the sexual response cycle[30]. These comprised the amygdala, anterior cingulate, caudate, globus pallidus, hippocampus, insula, nucleus accumbens, posterior cingulate, putamen, and thalamus.

Penile tumescence data acquisition and processing

As a validated modality for assessing sexual arousal in an fMRI environment[22], simultaneous penile tumescence was recorded continuously during the long video task and fMRI data acquisition using a custom-developed modular system. Before entering the MRI scanning room, participants donned an MR-compatible penile plethysmograph transducer (BioPac Systems, UK) worn around the midshaft of the penis. The liquid gallium transducer was connected to a bioinstrumentation amplifier (BioPac Systems, UK) via MEMRI-TRANS filtered cables, with the amplifier powered by a standard 12VDC switch-mode power supply in the control room, which was in turn powered through a KeyMed medical isolation transformer. The analogue signal that increased with increasing tumescence from the system was recorded by means of a standard data-acquisition system (PowerLab, AD Instruments, New Zealand) for off-line data analysis. At screening, each participant was familiarised with the device. To ensure correct fit of the

© 2023 Mills EG et al. *JAMA Network Open.*

transducer and accurate data collection, participants provided a flaccid penile circumference measurement using standardised instructions.

Data from the penile tumescence device recorded during the long video scan was processed using custom MATLAB (R2015b; Mathworks Ltd.) code. First, the data was trimmed to the correct length using the scanner TR pulse signal (also recorded on a separate channel) to identify the portions of the time-series that occurred during the scan period. Then the high-resolution (1000Hz) time-series was down-sampled and interpolated to match the temporal resolution (0.8Hz/1.25s) of the scan volume acquisition time (TR) and the number of volumes acquired (392). Finally, the time-series was smoothed with a simple moving-average filter (24 sample width) in order to reduce high-frequency noise[36]. This process produced a de-noised time-series of the correct sample rate and dimensions for inclusion in the fMRI analysis models. For the purposes of statistical analysis, data from each participants' time-series was also binned into 20 equal sections of 24 samples each; 20 bins were selected to provide a robust number of degrees of freedom for the analysis, while still preserving the temporal characteristics of the responses. Penile tumescence data was not obtained for two participants, due to malposition of the device by the participant and/or movement during the scan.

eReferences

1. Johnstone LT, Karlsson EM, Carey DP. Left-Handers Are Less Lateralized Than Right-Handers for Both Left and Right Hemispheric Functions. *Cerebral Cortex*. 2021;31(8):3780-3787.
2. American Psychiatric Association. *American Psychiatric Association: Diagnostic and Statistical Manual of Mental Disorders Fifth Edition*.; 2013.
3. Derogatis L, Rosen RC, Goldstein I, Werneburg B, Kempthorne-Rawson J, Sand M. Characterization of Hypoactive Sexual Desire Disorder (HSDD) in Men. *J Sex Med*. 2012;9(3):812-820.
4. Rosen RC, Riley A, Wagner G, Osterloh IH, Kirkpatrick J, Mishra A. The international index of erectile function (IIEF): a multidimensional scale for assessment of erectile dysfunction. *Urology*. 1997;49(6):822-830.
5. Dhillo WS, Chaudhri OB, Patterson M, et al. Kisspeptin-54 Stimulates The Hypothalamic-Pituitary Gonadal Axis In Human Males. *Journal of Clinical Endocrinology and Metabolism*. 2005;90(12):6609-6615.
6. Bjork JM, Gilman JM. The effects of acute alcohol administration on the human brain: insights from neuroimaging. *Neuropharmacology*. 2014;84:101-110.
7. Yang HC (Shawn), Liang Z, Yao J (Fiona), Shen X, Frederick B de B, Tong Y. Vascular effects of caffeine found in BOLD fMRI. *J Neurosci Res*. 2019;97(4):456.
8. Fox CA, Ismail AA, Love DN, Kirkham KE, Loraine JA. Studies on the relationship between plasma testosterone levels and human sexual activity. *J Endocrinol*. 1972;52(1):51-58.
9. Masters WH, Johnson VE. *Human Sexual Response*. 1st ed. Little, Brown & Company; 1966.
10. Georgiadis JR, Kringelbach ML. The human sexual response cycle: Brain imaging evidence linking sex to other pleasures. *Prog Neurobiol*. 2012;98(1):49-81.
11. Comninos AN, Wall MB, Demetriou L, et al. Kisspeptin modulates sexual and emotional brain processing in humans. *J Clin Invest*. 2017;127(2):709-719.
12. Jayasena CN, Nijher GMK, Comninos AN, et al. The Effects of Kisspeptin-10 on Reproductive Hormone Release Show Sexual Dimorphism in Humans. *Journal of Clinical Endocrinology and Metabolism*. 2011;96(12):e1963-72.
13. Comninos AN, Demetriou L, Wall MB, et al. Modulations of Human Resting Brain Connectivity by Kisspeptin Enhance Sexual and Emotional Functions. *JCI Insight*. 2018;3(20):e121958.
14. Yang L, Demetriou L, Wall MB, et al. Kisspeptin enhances brain responses to olfactory and visual cues of attraction in men. *JCI Insight*. 2020;5(3):e133633.
15. Yang L, Demetriou L, Wall MB, et al. The Effects of Kisspeptin on Brain Response to Food Images and Psychometric Parameters of Appetite in Healthy Men. *J Clin Endocrinol Metab*. 2020;106(4):e1837-e1848.
16. Comninos AN, Yang L, O'Callaghan J, et al. Kisspeptin modulates gamma-aminobutyric acid levels in the human brain. *Psychoneuroendocrinology*. 2021;129:105244.
17. Toledano R, Pfaus J. The sexual arousal and desire inventory (SADI): A multidimensional scale to assess subjective sexual arousal and desire. *Journal of Sexual Medicine*. 2006;3(5):853-877. d
18. Arnow BA, Millheiser L, Garrett A, et al. Women with hypoactive sexual desire disorder compared to normal females: A functional magnetic resonance imaging study. *Neuroscience*. 2009;158(2):484-502.
19. Thurston L, Hunjan T, Mills EG, et al. Melanocortin 4 receptor agonism enhances sexual brain processing in women with hypoactive sexual desire disorder. *J Clin Invest*. 2022;132(19).

© 2023 Mills EG et al. *JAMA Network Open.*

20. Thurston L, Hunjan T, Ertl N, et al. Effects of Kisspeptin Administration in Women With Hypoactive Sexual Desire Disorder: A Randomized Clinical Trial. *JAMA Netw Open*. 2022;5(10):e2236131.
21. Demetriou L, Kowalczyk OS, Tyson G, Bello T, Newbould RD, Wall MB. A comprehensive evaluation of increasing temporal resolution with multiband-accelerated protocols and effects on statistical outcome measures in fMRI. *Neuroimage*. 2018;176:404-416.
22. Ferretti A, Caulo M, del Gratta C, et al. Dynamics of male sexual arousal: Distinct components of brain activation revealed by fMRI. *Neuroimage*. 2005;26(4):1086-1096.
23. Moulier V, Mouras H, Pélégrini-Issac M, et al. Neuroanatomical correlates of penile erection evoked by photographic stimuli in human males. *Neuroimage*. 2006;33(2):689-699.
24. Friston KJ, Worsley KJ, Frackowiak RSJ, Mazziotta JC, Evans AC. Assessing the significance of focal activations using their spatial extent. *Hum Brain Mapp*. 1994;1(3):210-220.
25. Nichols TE. Multiple testing corrections, nonparametric methods, and random field theory. *Neuroimage*. 2012;62(2):811-815.
26. Eklund A, Nichols TE, Knutsson H. Cluster failure: Why fMRI inferences for spatial extent have inflated false-positive rates. *Proc Natl Acad Sci U S A*. 2016;113(28):7900-7905.
27. Redouté J, Stoléru S, Grégoire MC, et al. Brain processing of visual sexual stimuli in human males. *Hum Brain Mapp*. 2000;11(3):162-177.
28. Stoléru S, Redouté J, Costes N, et al. Brain processing of visual sexual stimuli in men with hypoactive sexual desire disorder. *Psychiatry Res*. 2003;124(2):67-86.
29. Mitricheva E, Kimura R, Logothetis NK, Noori HR. Neural substrates of sexual arousal are not sex dependent. *PNAS*. 2019;116(31):15671-15676.
30. Stoléru S, Fonteille V, Cornélis C, Joyal C, Moulier V. Functional neuroimaging studies of sexual arousal and orgasm in healthy men and women: a review and meta-analysis. *Neurosci Biobehav Rev*. 2012;36(6):1481-1509.
31. Kühn S, Gallinat J. A Quantitative Meta-Analysis on Cue-Induced Male Sexual Arousal. *J Sex Med*. 2011;8(8):2269-2275.
32. Mouras H, Stoléru S, Bittoun J, et al. Brain processing of visual sexual stimuli in healthy men: a functional magnetic resonance imaging study. *Neuroimage*. 2003;20(2):855-869.
33. Muir AI, Chamberlain L, Elshourbagy NA, et al. AXOR12, a Novel Human G Protein-Coupled Receptor, Activated by the Peptide KiSS-1. *Journal of Biological Chemistry*. 2001;276(31):28969-28975.
34. Kotani M, Detheux M, Vandenbogaerde A, et al. The metastasis suppressor gene KiSS-1 encodes kisspeptins, the natural ligands of the orphan G protein-coupled receptor GPR54. *J Biol Chem*. 2001;276(37):34631-34636.
35. Ohtaki T, Shintani Y, Honda S, et al. Metastasis Suppressor Gene KiSS-1 Encodes Peptide Ligand of a G-protein-coupled Receptor. *Nature*. 2001;411(6837):613-617.
36. Kettunen J, Ravaja N, Keltikangas-Järvinen L. Smoothing facilitates the detection of coupled responses in psychophysiological time series. *J Psychophysiol*. 2000;14(1):1-10.

© 2023 Mills EG et al. *JAMA Network Open.*

eFigure 1. Participant Recruitment and Flow Summary

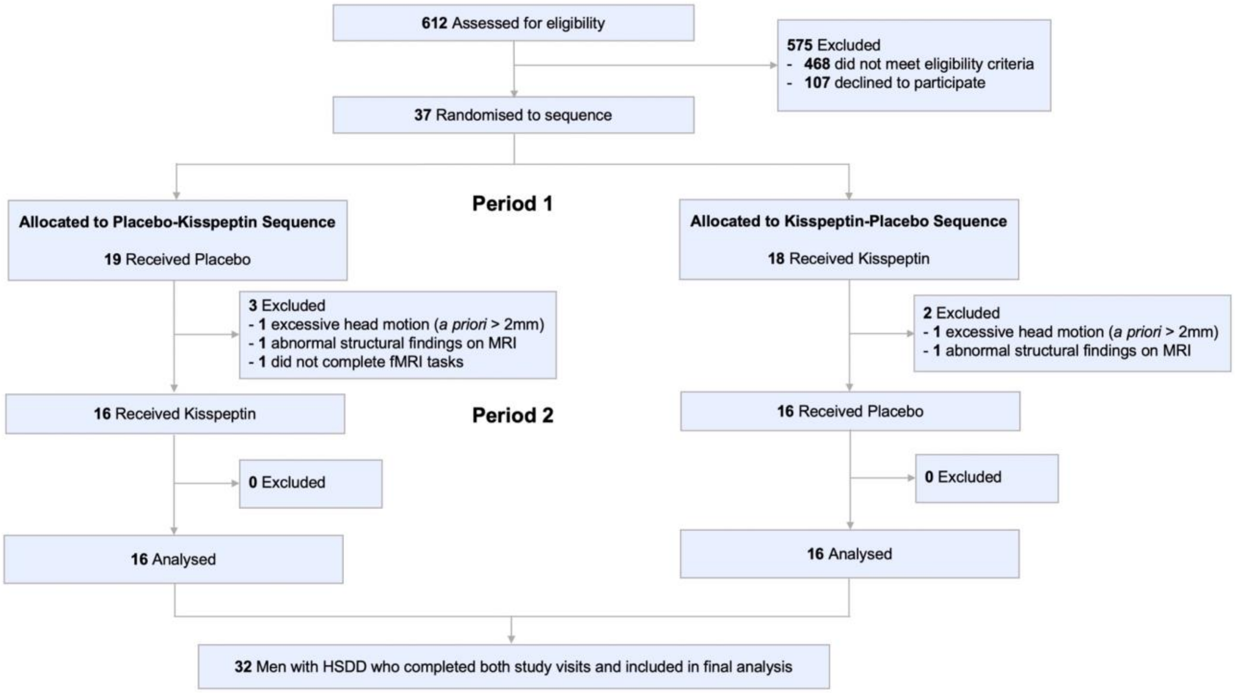

Per-protocol analysis included all men with Hypoactive Sexual Desire Disorder (HSDD), who appropriately completed both kisspeptin and placebo study visits (final *n*=32).

© 2023 Mills EG et al. *JAMA Network Open.*

eFigure 2. Effects of Kisspeptin Administration on Hormone Levels

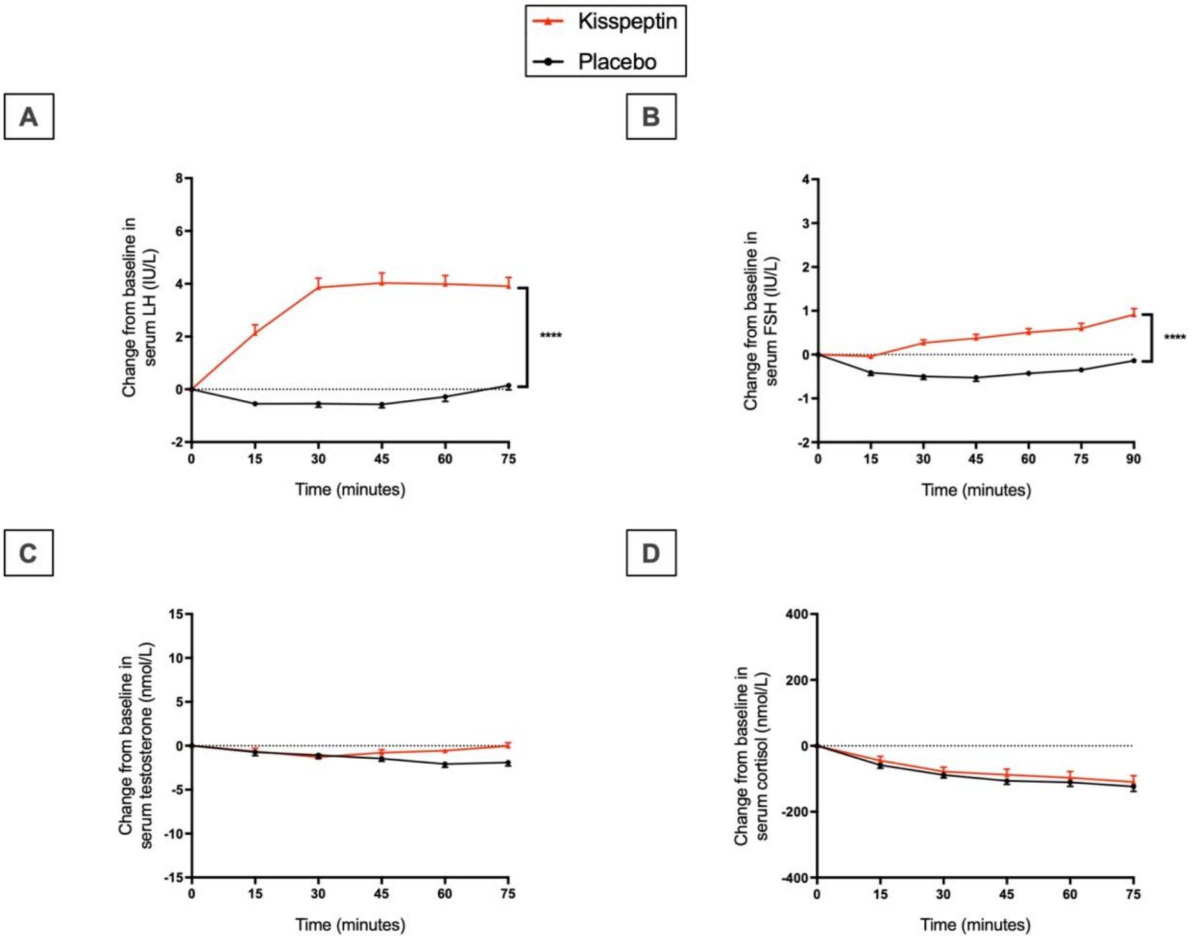

Kisspeptin administration significantly increased **(A)** circulating LH levels (mean difference (95% CI) = 3.29 IU/L (2.77,3.81), *P* <.0001) and **(B)** FSH levels (mean difference (95% CI) = 0.71 IU/L (0.55,0.88), *P* <.0001), but had no effect on **(C)** circulating testosterone or **(D)** cortisol levels**.** Data depict mean ± SEM. ****P <.0001, two-way ANOVA, n = 32.

© 2023 Mills EG et al. *JAMA Network Open.*

eFigure 3. Effects of Kisspeptin Administration on Sexual Arousal and Desire Inventory Scores

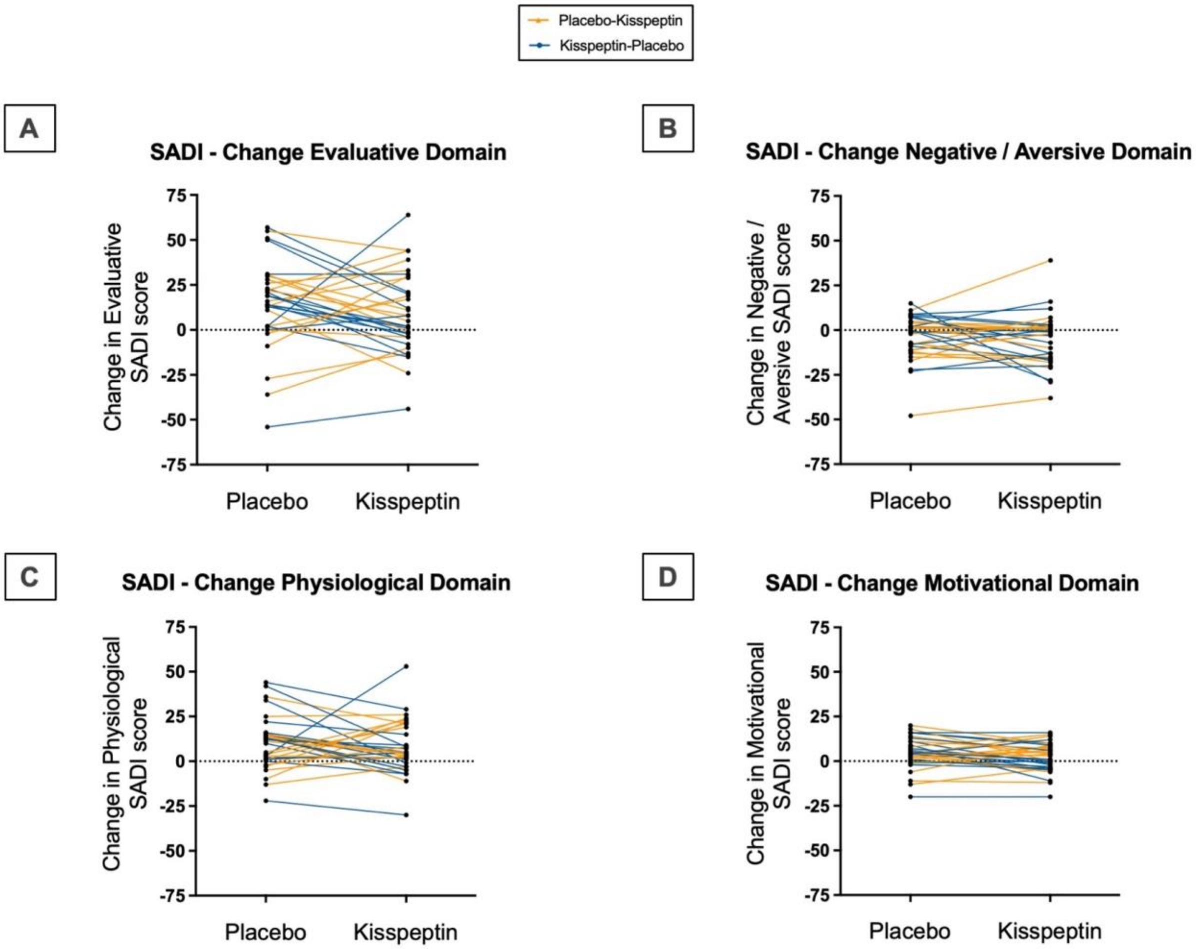

Sexual arousal and desire were assessed during kisspeptin and placebo administration using the SADI. Kisspeptin administration did not alter combined **(A)** evaluative; **(B)** negative/aversive; **(C)** physiological; **(D)** motivational domains. Presented as score change from baseline for each participant, *n* = 32. Orange line depicts participants who received placebo at first study visit and kisspeptin at second study visit (*n*=16). Blue line depicts participants who received kisspeptin at first study visit and placebo at second study visit (*n*=16). Total *n*=32.

© 2023 Mills EG et al. *JAMA Network Open.*

eFigure 4. Effects of Kisspeptin Administration on Positive and Negative Mood, Anxiety, and Nonsexual Attention

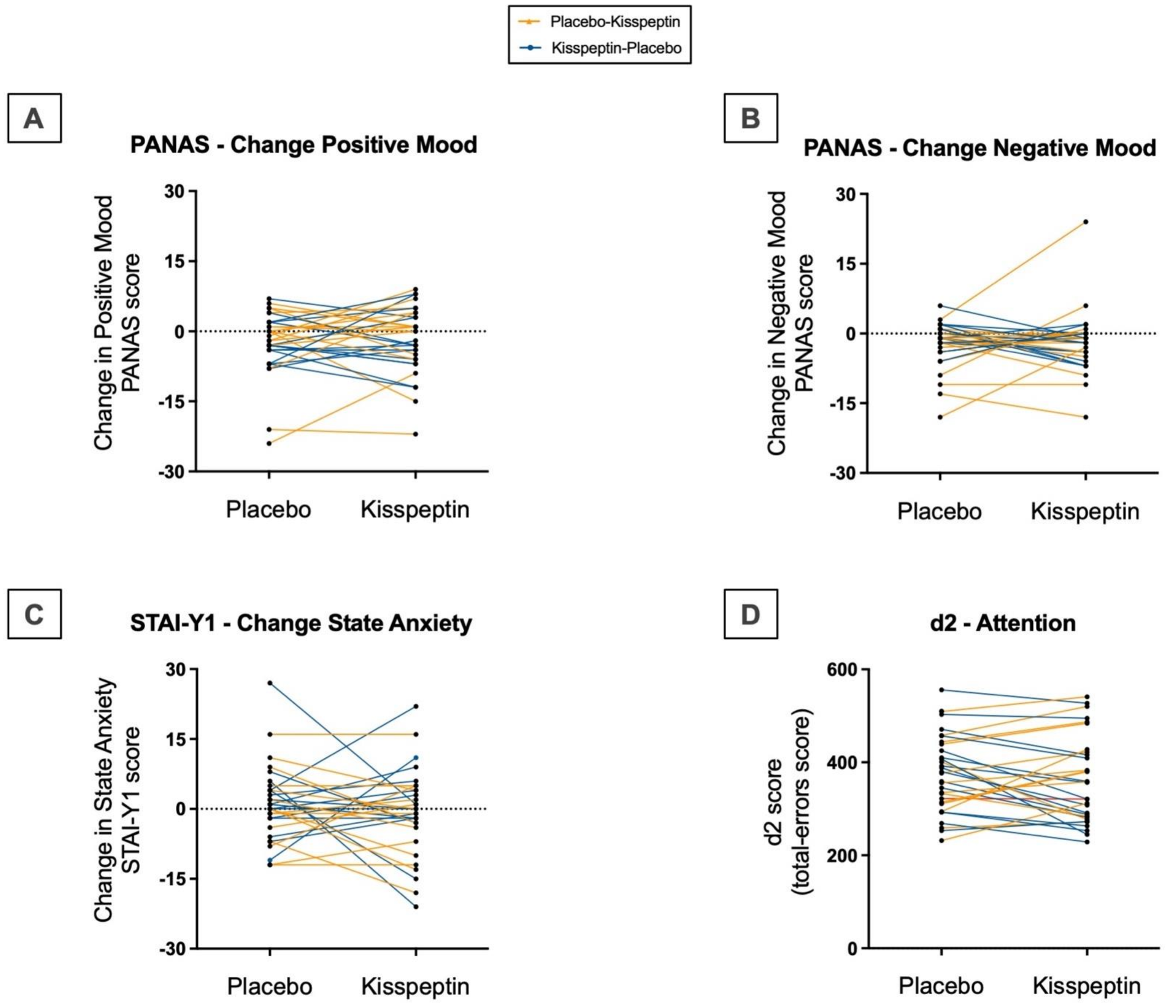

Positive and negative mood were assessed during kisspeptin and placebo administration using the Positive and Negative Affect Schedule (PANAS). Kisspeptin administration did not alter **(A)** positive mood; **(B)** negative mood. Presented as score change from baseline for each participant. **(C)** State anxiety before and during kisspeptin and placebo administration was assessed using the State-Trait Anxiety Inventory (State component, Form Y) and was unaltered by kisspeptin administration, compared to placebo. Presented as score change from baseline for each participant. **(D)** Non-sexual attention was assessed using the d2 test during kisspeptin and placebo administration and was unaltered by kisspeptin administration, compared to placebo. *n* = 32. Orange line depicts participants who received placebo at first study visit and kisspeptin at second study visit (*n*=16). Blue line depicts participants who received kisspeptin at first study visit and placebo at second study visit (*n*=16). Total *n*=32.

© 2023 Mills EG et al. *JAMA Network Open.*

eFigure 5. Effects of Kisspeptin Administration on Blood Pressure and Heart Rate Measurements

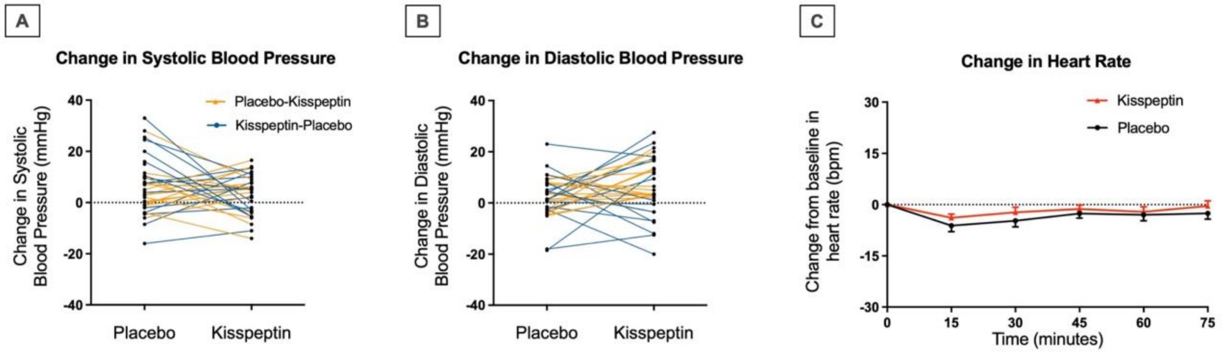

Blood pressure was assessed before and during kisspeptin administration. Kisspeptin administration had no significant effects on systolic **(A)** or diastolic **(B)** measurements, compared to placebo. Presented as change from baseline for each participant. Orange line depicts participants who received placebo at first study visit and kisspeptin at second study visit (*n*=16). Blue line depicts participants who received kisspeptin at first study visit and placebo at second study visit (*n*=16). **(C)** Heart rate was measured before and at 15-minute intervals during kisspeptin and placebo administration and was unaltered by kisspeptin administration, compared to placebo. Data depict mean ± SEM. *n* = 32.

© 2023 Mills EG et al. *JAMA Network Open.*

eFigure 6. Group Mean Analysis for Main Effects of the Short Videos Task in All Participants and Both Treatments (Kisspeptin and Placebo) Demonstrating Effective Task Design

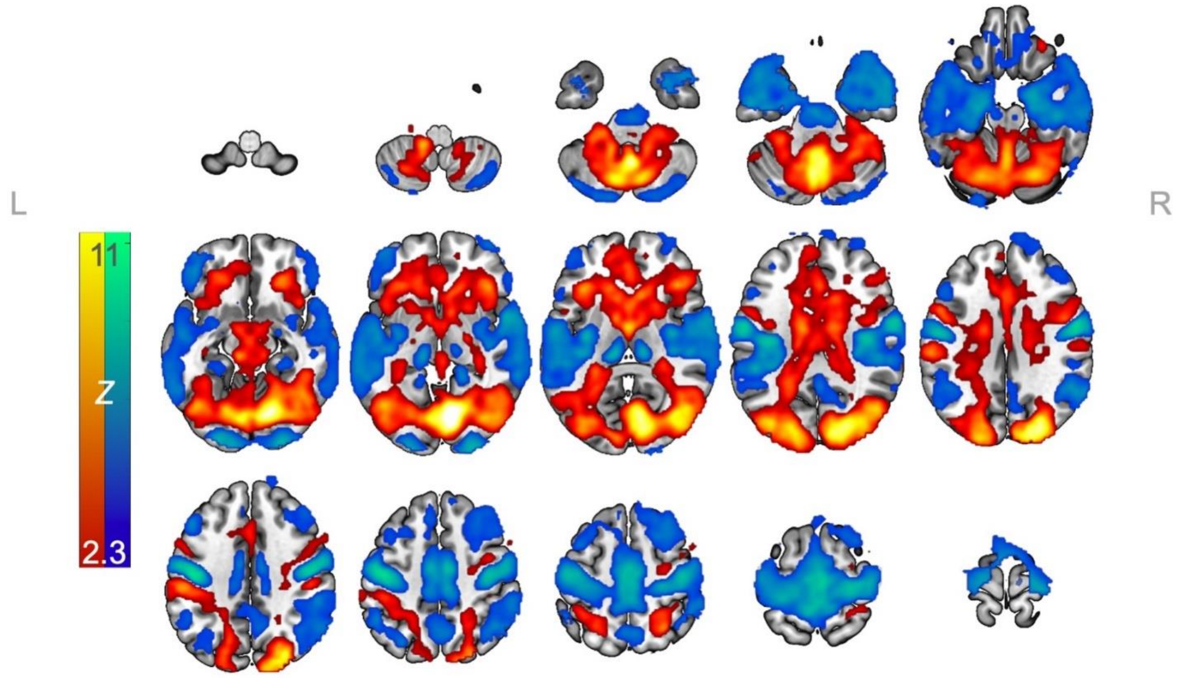

Red/yellow areas show increased activation to sexual compared to exercise videos (control). Blue/green areas show decreased activation to sexual compared to exercise videos (control). Clusters corrected for multiple comparisons, $Z = 2.3$, $P < .05$, $n = 32$.

© 2023 Mills EG et al. *JAMA Network Open.*

eFigure 7. Group Mean Analysis for Brain Areas Associated With Increasing Penile Tumescence During the Long Video Task in All Participants and Both Treatments (Kisspeptin and Placebo) Demonstrating Effective Task Design

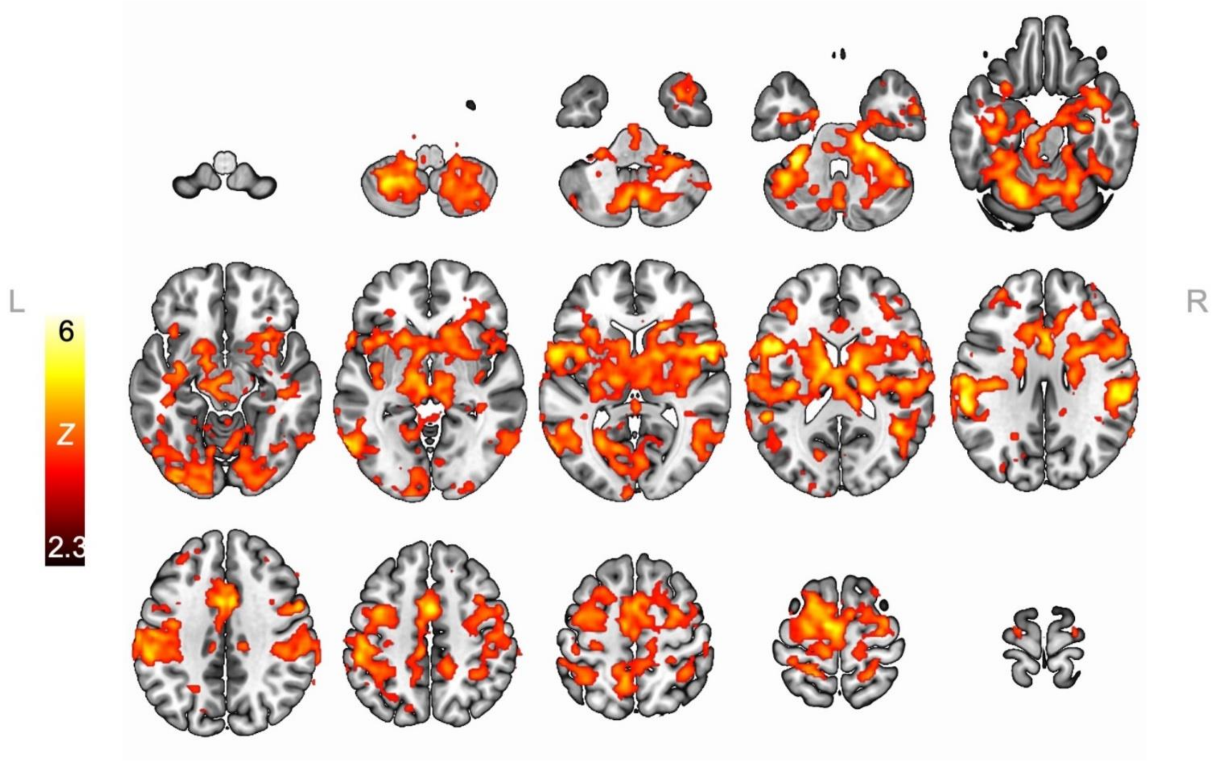

Red/yellow areas show increased activation as penile tumescence increased. Clusters corrected for multiple comparisons, $Z = 2.3$, $P < .05$, $n = 30$.

© 2023 Mills EG et al. *JAMA Network Open.*

eFigure 8. Group Mean Analysis of Brain Areas Associated With Increasing Subjective Arousal During the Long Video Task in All Participants and Both Treatments (Kisspeptin and Placebo) Demonstrating Effective Task Design

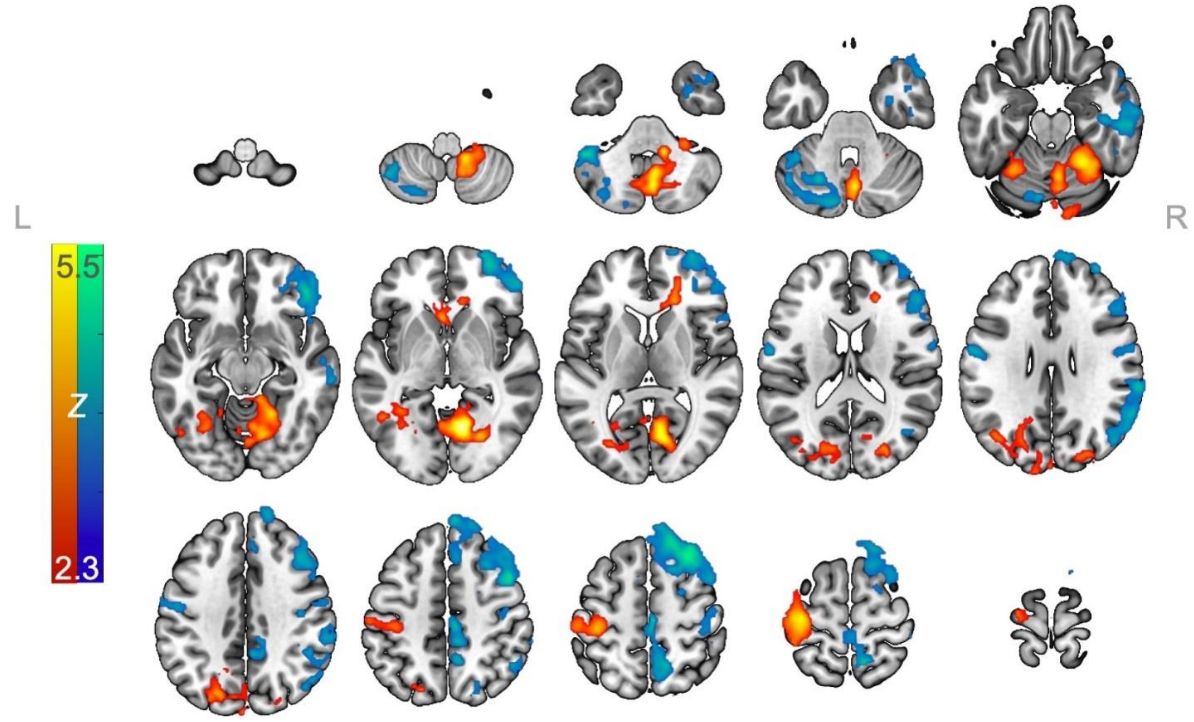

Red/yellow areas show increased activation as subjective arousal (determined by participants turning the scroll wheel) increased. Blue/green areas show decreased activation as subjective arousal increased. Clusters corrected for multiple comparisons, $Z = 2.3$, $P < .05$, $n = 32$.

© 2023 Mills EG et al. *JAMA Network Open.*

eTable 1. Participant Baseline Clinical and Psychometric Characteristics at the Beginning of the Kisspeptin and Placebo Visits

		**Kisspeptin Visit (*n* = 32)**	**Placebo Visit (*n* = 32)**
		Mean ± SD	Mean ± SD
**Baseline Hormones**			
	Kisspeptin (pmol/L)	22.6 ± 27.1	26.8 ± 21.2
	LH (IU/L)	3.1 ± 1.2	2.9 ± 1.2
	FSH (IU/L)	3.8 ± 2.7	3.8 ± 2.7
	Testosterone (nmol/L)	20.1 ± 5.5	19.1 ± 6.3
	Cortisol (nmol/L)	374.0 ± 111.5	363.1 ± 115.4
**PANAS** [a]			
	Positive affect	28.6 ± 7.8	27.2 ± 8.8
	Negative affect	16.7 ± 6.7	16.3 ± 6.6
**SADI** [b]			
	Evaluative	39.5 ± 24.9	32.9 ± 23.1
	Negative/aversive	22.2 ± 18.1	21.0 ± 16.8
	Physiological	18.9 ± 16.0	15.4 ± 15.0
	Motivational	15.1 ± 9.6	11.8 ± 8.9
**STAI-Y State** [c]		38.5 ± 9.8	36.5 ± 9.1

Abbreviations: LH = Luteinising Hormone; PANAS = Positive and Negative Affect Schedule; SADI = Sexual Arousal and Desire Inventory; STAI-Y State = State-Trait Anxiety Inventory-Form Y.

[a] PANAS: Scores range 10-50, with higher positive scores and lower negative scores indicating higher levels of positive affect and lower levels of negative affect, respectively.
[b] SADI: Evaluative scores range 0-135, negative aversive 0-85, physiological 0-85 and motivational 0-50.
[c] STAI-Y State: Scores range 20-80, with higher scores indicating higher levels of anxiety.
Sixteen participants received kisspeptin at first study visit and 16 participants received placebo at first study visit (double-blinded). Data presented as mean ± SD. *n* = 32.

© 2023 Mills EG et al. *JAMA Network Open.*

eTable 2. Clusters With Enhanced Activation or Deactivation by Kisspeptin on Whole-Brain Analysis

		(COG)	X	Y	Z	*P*	K	Z max
**Short videos task (Sexual > Control)**								
Anterior cingulate extending to Middle frontal gyrus	Activation		-20.4	20.9	31.9	.001	696	3.72
Parahippocampus	Deactivation		-7.48	-54.6	1.14	.007	566	4
**Long video task (subjective arousal)**								
Precuneus extending to Posterior cingulate	Deactivation		1.79	-21.8	56.4	.001	693	3.83
Frontal pole			-28.6	58.3	3.92	.044	383	3.6
**Long video task (penile tumescence)**								
Visual cortex and fusiform gyrus	Activation		12.3	-85.5	-2.09	<.001	1924	4.11

Data derived from whole brain analysis during short and long sexual video tasks (using regressors derived from subjective arousal and penile tumescence data). Coordinates represented by x (sagittal), y (axial) and z (coronal) and are derived from the MNI152 stereotactic coordinate space. Coordinates represent the centre of gravity (COG) for discrete activation and deactivation clusters observed in the group-level analyses of treatment effects (kisspeptin vs placebo), with statistical maps thresholded at Z = 2.3, *P* <.05 (cluster-corrected for multiple comparisons). K refers to the number of clusters, *p* shows the *p*-value for that cluster and Z max shows the maximum Z value of that cluster. *n* = 32 (except for penile tumescence where *n* = 30).

eTable 3. Effect Sizes for Brain Regions With Significantly Enhanced Activation or Deactivation by Kisspeptin Administration

								95% CI	
			**Statistic**	**d.f.**	***P***		**Effect size**	**Lower**	**Upper**
**Short videos task (Sexual > Control)**									
Anterior cingulate	Activation	**Paired t-test**	4.34	31	.001	**Cohen's *d***	0.77	0.37	1.16
Middle frontal gyrus	Activation	**Paired t-test**	4.66	31	.001	**Cohen's *d***	0.82	0.42	1.22
Parahippocampus	Deactivation	**Paired t-test**	-4.76	31	.007	**Cohen's *d***	-0.84	-1.24	-0.43
**Long video task (subjective arousal)**									
Frontal pole	Deactivation	**Paired t-test**	-2.62	31	.044	**Cohen's *d***	-0.46	-0.82	-0.09
Posterior cingulate	Deactivation	**Paired t-test**	-3.31	31	.001	**Cohen's *d***	-0.59	-0.96	-0.21
Precuneus	Deactivation	**Paired t-test**	-2.38	31	.001	**Cohen's *d***	-0.42	-0.78	-0.06
**Long video task (penile tumescence)**									
Fusiform gyrus	Activation	**Paired t-test**	1.47	29	<.001	**Cohen's *d***	0.27	0.10	0.63
Visual cortex	Activation	**Paired t-test**	2.76	29	<.001	**Cohen's *d***	0.50	0.12	0.88

Data derived from whole brain analysis during short and long sexual video tasks (using regressors derived from subjective arousal and penile tumescence data). To test for differences between kisspeptin and placebo administration, a within-subjects paired *t*-test general linear model was constructed. To examine task main effects an additional group average model was constructed which averaged across both drug conditions. Cohen's *d* was used as an effect size statistic for the paired *t*-test. 95% CI refers to the 95% confidence interval, d.f. to number of degrees of freedom and *p* shows the *p*-value for that brain region. *n* = 32 (except for penile tumescence where *n* = 30).

© 2023 Mills EG et al. *JAMA Network Open*

eTable 4. Safety Outcomes

		**Kisspeptin Visit (*n* = 32)**	**Placebo Visit (*n* = 32)**	**Total (*n* = 32)**
**Number of participants with at least 1 AE**		0	0	0
Cardiac disorders	Tachycardia	0	0	0
Gastrointestinal disorders	Nausea	0	0	0
	Vomiting	0	0	0
	Abdominal pain	0	0	0
	Diarrhoea	0	0	0
General disorders	Procedural pain	0	0	0
	Pyrexia	0	0	0
	Chills	0	0	0
	Hypotension	0	0	0
	Hypertension	0	0	0
	Decreased appetite	0	0	0
Musculoskeletal disorders	Back pain	0	0	0
	Arthralgia	0	0	0
	Myalgia	0	0	0
Nervous system disorders	Headache	0	0	0
	Dizziness	0	0	0
Respiratory disorders	Cough	0	0	0
	Dyspnoea	0	0	0
Skin disorders	Pruritus	0	0	0
	Rash	0	0	0
	Erythema	0	0	0
**Number of participants with at least 1 SAE**		0	0	0
**AEs leading to discontinuation of study**		0	0	0
**Study-related AEs leading to discontinuation of study**		0	0	0

Abbreviations: AE = Adverse Event; SAE = Serious Adverse Event.
Data represents the number of participants experiencing any AE or SAE and the number of participants experiencing any AE leading to discontinuation of study. *n* = 32.

© 2023 Mills EG et al. *JAMA Network Open*
