# Supplementary material for: Effects of Kisspeptin on Sexual Brain Processing and Penile Tumescence in Men With Hypoactive Sexual Desire Disorder: A Randomized Clinical Trial
Source: JAMA Netw Open. 2023 Feb 3;6(2):e2254313. doi: 10.1001/jamanetworkopen.2022.54313 (PMC9898824; doi:10.1001/jamanetworkopen.2022.54313)
Supplement: Supplement 3. — Data Sharing Statement [file jamanetwopen-e2254313-s003.pdf]

Data Sharing Statement

Mills. Effects of Kisspeptin on Sexual Brain Processing and Penile Tumescence in Men With Hypoactive Sexual Desire Disorder. *JAMA Netw Open*. Published February 03, 2023. doi:10.1001/jamanetworkopen.2022.54313

Data
Data available: No

Additional Information
Explanation for why data not available: Some or all data sets generated during and/or analyzed are not publicly available but available from the corresponding authors on the basis of reasonable scientific merit. All data provided are anonymised to respect the privacy of the participants. Custom Matlab code used to process the penile plethysmograph data is available from author MBW on reasonable request.
